# PAH Induction upon Pyrolysis of Hydroxyl-Terminated Polybutadiene-Based Solid Rocket Fuels

**DOI:** 10.1021/acs.jpca.5c03340

**Published:** 2025-07-07

**Authors:** Valeriia Karpovych, Nataliia Haiduk, Eugene Oga, Nafisa Bala, Alena Kubátová, Evguenii Kozliak, Mark Sulkes

**Affiliations:** 1 Department of Chemistry, 5783 Tulane University, New Orleans, Louisiana 70118, United States; 2 Department of Chemistry, 3579 University of North Dakota, Grand Forks, North Dakota 58202, United States

## Abstract

Using a molecular beam (MB)-based approach with time-of-flight mass spectra (TOFMS) product detection, we have effected pyrolysis of hydroxyl-terminated polybutadiene/ammonium perchlorate (HTPB/AP) mixtures under conditions relevant to solid fuel rocket motor operation – pyrolysis temperatures >1000 °C with chemistry occurring on μs time scales. In earlier studies, it was implicitly assumed that AP did not significantly affect HTPB pyrolysis chemistry, that it was a featureless oxidant that also raised pyrolysis temperatures. This appears not to be the case. For HTPB pyrolysis in the presence of AP, a series of polycyclic aromatic hydrocarbon (PAH) products were detected up to *m*/*z* 240–260. No such PAHs were detected for pyrolysis of HTPB alone, with traces of them occurring when HTPB was cross-linked with methylene diphenyl diisocyanate. The presence of AP also resulted in the concomitant induction of cyclic C_6_ products with *m*/*z* 76 and 78, benzyne and benzene, at the expense of acyclic C_6_ product peaks at *m*/*z* 83–84 (presumably, an inhomogeneous distribution of hexenes and the corresponding hexenyl radicals) that were predominant in the absence of AP. The occurrence of PAH peaks was confirmed by evolved gas analysis with mass spectrometric detection (EGA-MS). The observation of similar *m*/*z* values, up to 240–260, when using short time scale MB and much longer time scale EGA-MS and pyrolysis-GC-MS (Pyr-GC-MS), indicates that the observed PAH growth occurs on a short time scale of tens of microseconds yielding the aromatic products with up to four and five rings. The use of Pyr-GC-MS enabled the identification of the exact PAH products and tentative pathways toward their formation.

## Graphical abstract

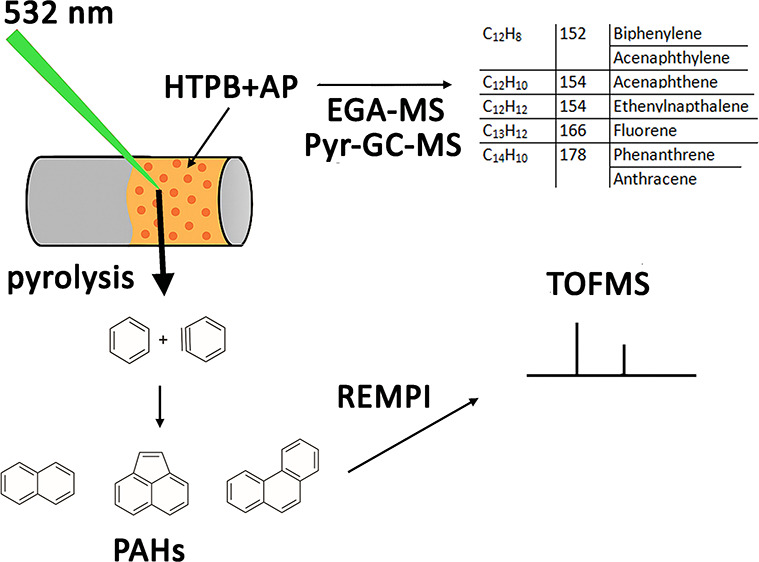

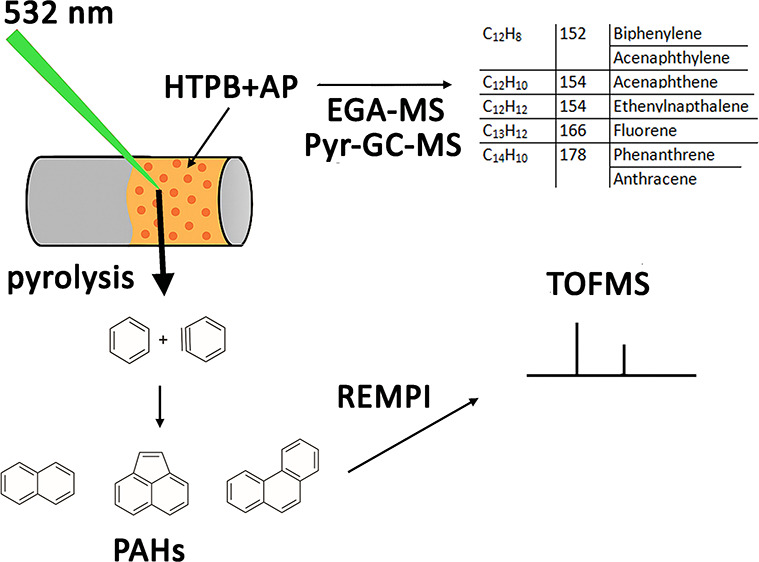

## Introduction

Hydroxyl-terminated polybutadiene (HTPB) is used worldwide as solid fuel and binder in rockets and ramjets, in mixtures with ammonium perchlorate (AP, NH_4_ClO_4_), aluminum powder and minor additives, e.g., ballistic modifiers.^1–3^ Typical solid rocket fuel consists primarily of submm AP particles suspended in a binder matrix consisting of diisocyanate cross-linked HTPB, often using methylene diphenyl diisocyanate (MDI) as a cross-linker.^2^

For accurate modeling of HTPB pyrolysis product combustion, knowledge of the actual chemical composition and speciation is essential.^1^ Without this knowledge, models must be based on simplified chemical speciation, using generic low-molecular weight (MW) species, e.g., ethylene, to represent the complex organic composition.^3,4^ To enable improvement in these models, a number of studies were conducted on the chemistry occurring during neat HTPB pyrolysis, focusing on product speciation.^5–9^ In operating rocket motors, relevant temperatures range from ∼600 °C at the burning solid fuel surface to ∼1200 °C at the AP monopropellant flame, a separation in distance of only microns.^1^ While temperatures in this range can be replicated by methods such as pyrolysis-gas chromatography with mass spectrometric detection (Pyr-GC-MS), the extremely steep thermal gradient inherent for combustion means that relevant heating rates can be as high as 10^6^ °C/s. Since the time scale for chemistry in the Pyr-GC-MS studies is in the seconds range, it is not evident that the products detected in those studies^5–7^ are actually relevant to combustion. Two studies using flash pyrolysis methods for faster heating were able to approach a heating rate of 10^3^ °C/s,^8,9^ still well short of the relevant rates. The foregoing studies were reported in the 1990s to 2000. A few subsequent experimental studies did not add any significantly new information, presumably because faster heating rates could not be attained.^10–12^

These slower methods fail to capture the chemistry occurring on true motor operation time scales, hence the need for our MB-based approach. We have recently repurposed standard supersonic gas expansion molecular beam (MB) methods such that they can be used to study pyrolysis product chemistry occurring on microsecond time scales,^13^ now with temperatures and also heating rates relevant to operating rocket motors. Having applied these methods to investigate neat HTPB pyrolysis product chemistry,^14^ it is not entirely surprising that the major product profiles we observed did not exactly match the ones obtained in the earlier studies, although generally, we detected similar products, species containing 2–7 carbon atoms. (It is likely that there were also C1 products, namely methane. However, its ionization potential of 12.6 eV^15^ is higher than that we can detect with 118 nm photons of 10.5 eV.) Unlike results from prior publications obtained in the 800–850 °C range, where benzene and toluene were detected as substantial products, we observed little or none of them. By contrast, several mechanistically important radical species were identified. It was also observed that they processed further in time, stabilized by both hydrogenation and dehydrogenation. Hydrocarbon species containing three carbon atoms were abundant compared to prior studies, at the expense of higher-MW products, particularly 4-vinylcyclohexene, which was observed in a number of prior studies.^5–9^ Thus, the knowledge gap in *primary* pyrolytic product identification was addressed. However, a knowledge gap still remains in terms of *secondary* pyrolytic products, those formed in association with chemical reactions. This study addresses that problem for aromatic species that we are able to detect using resonance enhanced multiphoton ionization (REMPI) methods on shorter time scales^16^ while using evolved gas analysis with mass spectrometric detection (EGA-MS) to extend the time scale to significantly longer values. Then, Pyr-GC-MS was used for accurate identification of the PAH products formed to decipher the chemical mechanism of their formation.

Furthermore, the prior studies generally did not consider the impact of AP and the cross-linking of HTPB. Most of these works investigated pyrolysis of neat HTPB, with the implicit assumption that AP is merely a featureless oxidant.^5,6,8,9^ Only the Pyr-GC-MS studies of Ganesh et al.^7^ and Brill and Budenz,^4^ conducted on much longer time scales, addressed the determination of HTPB plus AP decomposition products.^7^ Ganesh et al. observed several low-MW PAHs when AP was present – and none when it was absent. PAHs are well-known soot precursors and environmental pollutants. Besides environmental concerns, soot accumulation initiated by PAH formation would impact possible reusable launchers, thus making the investigation of PAH formation practically relevant.

In our initial study of HTPB pyrolysis products, we employed 118 nm single photon ionization (SPI) for general product detection.^14^ These products were predominantly aliphatic. For selective and sensitive detection of aromatic compounds (or just “arenes”), resonant two photon ionization is particularly useful,^16^ an instance of REMPI. Here, the first photon has a wavelength in the vicinity of excited state absorbing levels of the aromatic species. For this purpose, 266 nm photons, the fourth harmonic of Nd:YAG lasers, are conveniently available; a second 266 nm photon is sufficient to reach the ionization states. REMPI can be orders of magnitude more sensitive than a nonresonant two photon ionization process (i.e., for species with no absorbing levels in the vicinity of 266 nm, typically the case for saturated aliphatic hydrocarbons), making it ideal for tracking aromatic species. For MB-based pyrolysis studies, we have previously employed this REMPI method for the detection of PAHs formed at short residence times in several other systems, including pyrolysis of triglycerides, 2,4-dinitrotoluene and lignin.^17–22^

Herein, we extended our studies on neat HTPB to consider pyrolysis products of diisocyanate cross-linked HTPB, with and without the inclusion of AP, using REMPI for sensitive detection of aromatic compounds. In this paper, we are principally concerned with their influence on the formation of polycyclic aromatic products, particularly PAHs. Questions we have addressed herein are as follows.

*What controls whether PAHs form on microsecond time scales?*

1. Would any PAHs be observed on combustion relevant time scales of microseconds?
2. Is AP essential for PAH generation?

*How do formulation and pyrolysis duration impact PAH identity and complexity?*

1. Would the cross-linked HTPB, central to producing a solid fuel, generate more or less PAHs?
2. What could be the main PAH precursor and pathway of PAH formation?
3. How far would the PAH growth extend in terms of the number of aromatic rings per molecule when the time scale increases from microseconds to seconds when using EGA-MS and Pyr-GC-MS for their detection?

## Methods

### MB Methods: Repurposing to Study Pyrolysis Product Chemistry on Microsecond Time Scales

Supersonic gas expansion MB methods have long been employed to generate, detect, and study novel, usually transient, species.^22,23^ Key to that approach is the fact that target species transiently produced at the front end of rare gas expansions are rapidly cooled (μs time scales^13^) and placed in low collision environments. Traditional MB studies use REMPI methods with the detection of wavelength scanned transitions in a single *m*/*z* channel corresponding to the target species parent mass. Our approach effects pyrolysis at the front end of supersonic gas expansions via laser pulsed surface heating of samples deposited on the heated surfaces. Rare gas pulses entrain the pyrolyzed species and undergo supersonic gas expansion. At the conclusion of the gas expansions, a matter of microseconds, the pyrolyzed species in center stream have been internally cooled and now have negligible collision rates on microsecond time scales. This portion is skimmed to form a molecular beam. Rather than study a particular target species, we now carry out photoionization (PI) TOFMS to characterize the products present.

This approach allows for fast (ns) laser pulse surface heating, enabling observation and monitoring the pyrolysis chemistry occurring on the time scale of supersonic gas expansions, μs.^13^ As a result, early product chemistry, including radical species, can be accessed. However, the deficiency of MB mass-based detection is the impossibility of exact product (PAH) identification when isomers are present; therefore, the use of complementary methods, e.g., Pyr-GC-MS, is essential, even though they are conducted on a much longer time scale. This study uses a combination of these two methods, with the addition of EGA-MS, a “bridging” method that occurs on an intermediate time scale.

The MB experimental arrangement is schematized in Figure 1. It is the same one as used in our previous work,^13,14^ designed to study pyrolysis product chemistry. Systems to be pyrolyzed are deposited on *d* = 0.25″ ceramic rod surfaces. Neat HTPB, MDI cross-linked HTPB, and AP do not absorb at 532 nm. Therefore, an absorbing surface, or surface matrix, is needed over which HTPB depositions are made to be heated by the 532 nm laser pulses. Pyrolysis chemistry occurs beginning at the rod surface and extending ∼10 mm past the rod.

**Figure 1:**
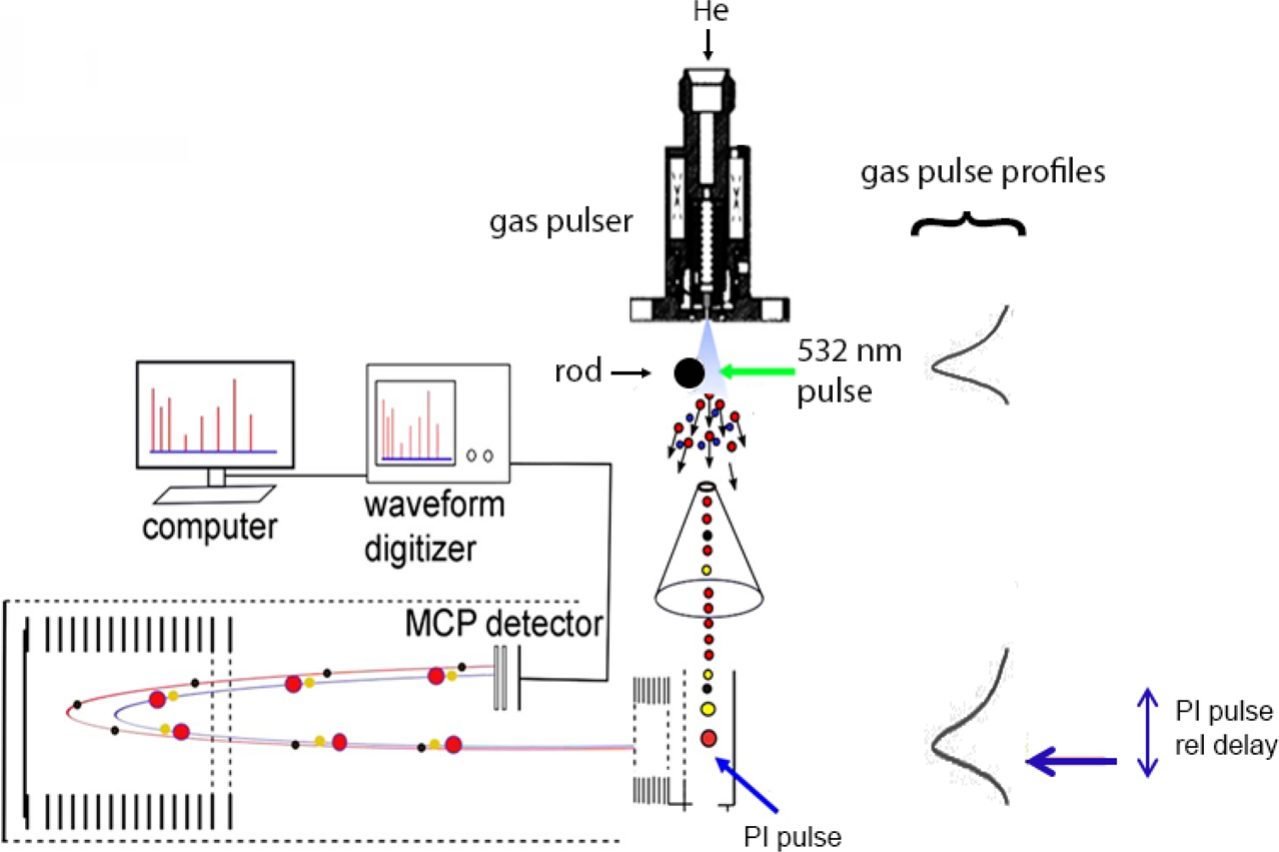
Experimental schematic of MB experiments. The 532 nm pulses heat the rod surface and initiate pyrolysis in the HTPB over that spot. Some of these products are entrained in the passing gas pulse. Note that later portions of the gas pulse also entrain products at the same point that now have had additional time for chemistry to occur before entrainment.

A particularly effective matrix for surface heating is graphite, with which we have obtained our strongest signals, i.e., highest signal-to-noise ratio. Our original procedure involved deposition of <45 μm graphite powder followed by HTPB sample deposition. We subsequently found that we could obtain results of the same quality by spraying a dry film graphite lubricant (Sprayon, LU204) directly onto ceramic rods. To identify any artificial features, e.g., extra peaks, due to the presence of graphite, we also used sandblasted tool steel, yellow brass, and aluminum rods on which HTPB preparations were deposited.

The sequence of experimental events, recorded at a rate of 10 Hz, was as follows: (1) The gas pulser opened (Jordan Time of Flight; fwhm of gas pulses ∼ 80 μs). Typical He gas backing pressures used were 175 psig. (2) Rods were placed ∼3–5 mm below the gas pulser. The 532 nm ablation (i.e., effective heating) pulses (second harmonic of Nd:YAG laser) were timed to hit the rod surface concurrent with the arrival of gas pulses. Focused spot size was *d* < 1 mm. Pyrolysis product peaks were typically obtained with ablation pulse energies ∼3–11 mJ. The rods were slowly rotated (several RPM) through a 40 tpi leadscrew, such that a fresh surface was presented for each 532 nm ablation pulse. (3) Each gas pulse entrained pyrolysis species with subsequent supersonic expansion, cooling internal degrees of freedom. A MB was formed by skimming the center stream of the gas pulses. (4) In the ion source region of the apparatus, PI pulses were timed to cross the molecular beam. For SPI, 118 nm pulses were generated by focusing ∼10–15 mJ 355 nm pulses in an Xe/Ar gas cell. For 266 nm REMPI, unfocused (beam diameter ≈ 2 mm) 266 nm pulses of ∼1–2 mJ were employed. In some circumstances, when no peaks were detected by this method, the 266 nm beam was focused.

Generally, the timing was set so that the PI pulses crossed near the front edge of the gas pulses, resulting in the strongest signals. However, the PI pulses could be further delayed so that they crossed further downstream portions of the gas pulses. In such instances, the relevant portion of the gas pulse – for example, 10 μs from the front edge – passed over the 532 nm ablation point 10 μs later. As a result, an additional 10 μs of chemistry processing time was afforded at the ablation point, resulting in possible additional chemistry prior to entrainment in the gas pulse.

Benzylpyridinium “thermometer” ion measurements can provide useful characterizations of pyrolysis product temperatures.^14,24^ In standalone experiments with He carrier gas where thermometer ions were deposited on a rod with a prior graphite deposition, front edge determined temperatures were found to vary linearly with 532 nm pulse energy.^24^ However, for different graphite surface preparations and other experimental details, the linear relation can vary. Temperatures attained may depend on the carrier gas used, backing pressure, and gas pulse shape. There is a strong dependence on details of the graphite deposition, possibly whether there was a prior deposition of boron nitride, and on the deposition thickness of graphite. In the context of HTPB pyrolysis, the most useful thermometer ion measurements have involved placement of HTPB on one portion of a graphite matrix rod with the thermometer ion on a different portion of the same rod. In such cases, it was possible to correlate the appearance of the *m*/*z* 83–84 peak with thermometer ion temperature. The appearance temperature for this peak was found to be close to 1200 K—low 900 °C.^25^ More substantial *m*/*z* 83 peaks, as observed in the TOFMS reported here, correspond to pyrolysis temperatures of 1000 °C or more.

HTPB pyrolysis products that were soft ionized by the PI pulses then underwent MS analysis in a reflectron based TOF system. Each pulsed sequence generated a TOFMS waveform. Typically, hundreds or more waveforms were recorded for good signal-to-noise results.

## Materials

For MB-TOFMS experiments, HTPB (−OH terminal groups; equivalent weight = 1250 Da), MDI, and AP were purchased from Rocket Motor Components (www.rocketmotorparts.com). Neat HTPB was solubilized in chloroform; mists were deposited on rods using an artist’s airbrush, from which the chloroform rapidly evaporated. For MDI cross-linked HTPB, an 87.1% HTPB and 12.9% MDI weight mixing ratio was used. Cure times were ∼6 days. Similar deposition methods were used for cross-linked HTPB. Subsequent deposition of AP could not be performed via dispersion in chloroform solutions. Instead, rods with deposited cross-linked HTPB were rolled in AP powder and placed as a uniform thin layer on a flat surface. For thinner depositions, AP was sprinkled on top of horizontally rotated HTPB prepared rods.

Before and after deposition, the rods were weighed. This allowed for the calculation of mean deposition weights per area. Neat and cross-linked HTPB depositions were typically 1–3 mg/cm^2^. The AP to HTPB weight ratios varied between ≈1 and 5.

For EGA-MS and Pyr-GC-MS, a quartz filter was placed in a deactivated stainless steel Eco cup (ID 3.8 mm, height 8 mm, wall thickness 0.1 mm, volume 80 μL) purchased from Frontier Laboratories (Ltd., Fukushima, Japan). The neat HTPB samples (∼200 mg) were loaded on the filter in the cup.

## EGA-MS and Pyr-GC-MS Experiments

To enable online pyrolysis experiments on a longer time scale, a PY-3030D multishot pyrolyzer with an autoshot sampler (Frontier Laboratories) was coupled to an Agilent 7890A GC system with a quadrupole 5975C MS detector (Agilent, Santa Clara, CA, USA). The experiments were conducted in two modes (i.e., EGA-MS and Pyr-GC-MS) as described below.

In the EGA-MS mode, the GC inlet was maintained at 300 °C in split mode (1:50) and the GC column was replaced with a deactivated tube (Ultra ALLOY-DTM, 0.15 mm ID × 3 m length, Frontier Laboratories) for direct analyte transfer from the pyrolyzer to the MS using helium as a carrier gas at a flow rate of 1.0 mL/min. Samples were isothermally pyrolyzed at 500, 600, 700, and 800 °C for 10 min each (the majority of the products evolved within 0.5 min). MS analysis was performed in full scan mode (10–700 *m*/*z*) using electron ionization (EI) at 70 eV with a rate of 7.2 s/scan.

The second mode, Pyr-GC-MS, was performed using a DB-5MS GC capillary column (32 m length, 0.25 mm ID, and 0.25 μm film thickness). Samples were pyrolyzed at 700 and 800 °C for 0.5 min each. The GC inlet was set to 300 °C in split mode (1:20), with helium as the carrier gas (1.0 mL/min). The GC oven temperature program started at 60 °C held for 1 min, followed by a ramp rate of 10 °C/min to 300 °C and held for 1 min, then 1 °C per minute to 320 °C and held at a final time of 4 min to enable sufficient separation of PAH isomers but for chrysene and triphenylene. MS analysis was conducted in full scan mode (10–700 *m*/*z*) using EI at 70 eV and a scan rate of 0.4 s/scan.

PAH identification was performed by matching retention times of some commercially available standards denoted as (S) and/or by matching the analyte MS spectrum to the NIST–2020 Library based on the percentage match denoted as T (% match). The minimum percent match observed was 70%.

## Results and Discussion

### 118 nm SPI: The Influence of AP and Cross-Linking on TOFMS Profiles

In contrast to our previous paper,^14^ which reported pyrolysis products of neat HTPB alone, herein we consider the case most relevant to solid rocket fuels, probing the pyrolytic products formed by MDI cross-linked HTPB with and without AP. The 118 nm SPI TOFMS of cross-linked HTPB with and without AP are shown in Figure 2B. The advantage of using 118 nm SPI is that it is nonselective; all products present should produce peaks, roughly in proportion to relative species population. With the exception of the new peaks at *m*/*z* 76 and 78 for the AP case, with a concomitant decrease in *m*/*z* 83–84 (all three being due to key C_6_ size products), the product peaks seen are similar in each case and also similar to the earlier observations of neat HTPB alone.^14^ The disadvantage of SPI is that aromatic peaks at *m*/*z* > 100 were barely discernible, if at all discernible; REMPI was used in the next sections to enhance them. In each experiment, there can be some rod preparation dependent differences (e.g., distribution of different C_4_ compound peaks; 1,3-butadiene was often, but not always, hydrogenated or dehydrogenated).

**Figure 2:**
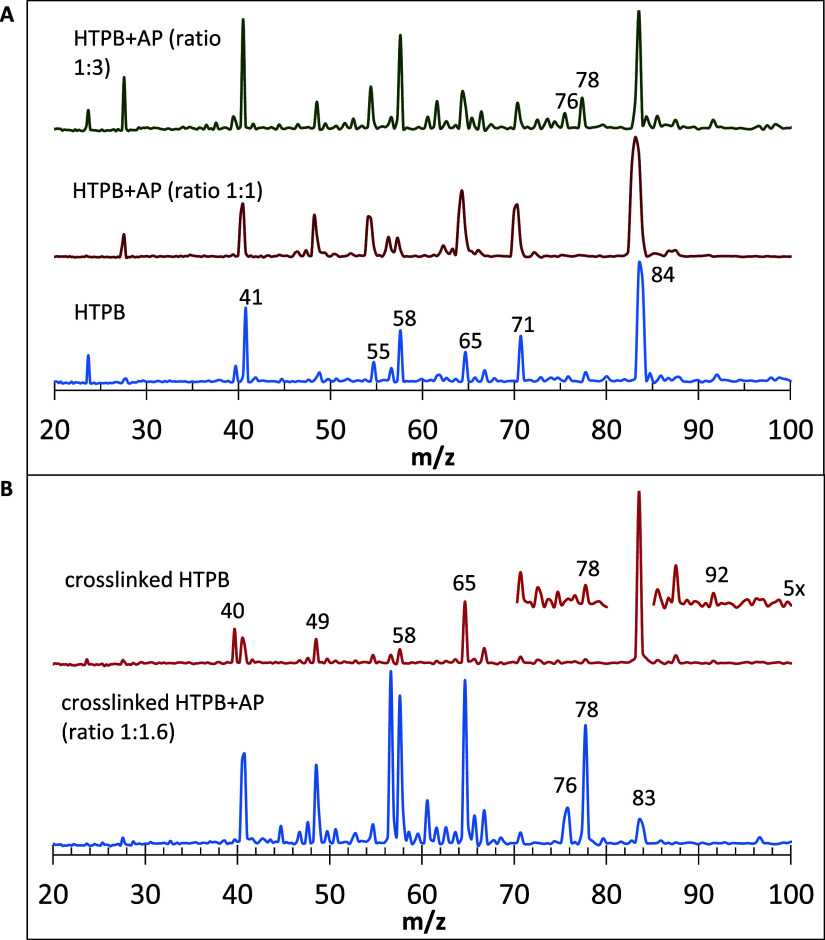
Effect of AP on pyrolysis products of HTPB, both cross-linked and neat. (A) 118 nm PI TOFMS with neat HTPB, then using two different HTPB:AP ratios. (B) 118 nm SPI TOFMS of MDI cross-linked HTPB versus MDI cross-linked HTPB with the addition of AP. A mixture of HTPB plus MDI was placed on a ceramic rod with the prior deposition of a graphite slurry; cross-linking subsequently occurred. AP was deposited on one portion of the rod. In each instance, 118 nm PI was conducted at the front edge of the He gas pulses.

Species of C_3_–C_6_ size appeared in 118 nm PI TOFMS (Figures 2 and 3), with peaks of ions produced by hydrocarbons and corresponding free radicals of a varied degree of unsaturation. The C_3_ species were represented with two major signals of *m*/*z* 40, C_3_H_4_ (either allene or propyne) and *m*/*z* 41, C_3_H_5_ (allyl radical). C_4_ products yielded a series of peaks ranging from *m*/*z* 49 (diacetylene radical) to 58 (butane, C_4_H_10_). C_5_ species also yielded a number of peaks, with a major one of *m*/*z* 65, C_5_H_5_, presumably the cyclopentadienyl radical. A peak at *m*/*z* 71, usually appearing on graphite but also sometimes observed on metal rods, is evidently due to a pentyl radical. Traces of C_7_ species were detected, represented by toluene, *m*/*z* 92. An interplay of hydrogenation and dehydrogenation reactions noted in our previous publication^14^ resulted in significant C=C bond hydrogenation of certain products, e.g., *m*/*z* 57 and 58 among the C_4_ species, at the expense of the other compounds, which were significantly dehydrogenated, e.g., most of C_5_ and some C_4_ products. A qualitatively similar primary pyrolytic product composition was reported in earlier studies.^5–9^

**Figure 3:**
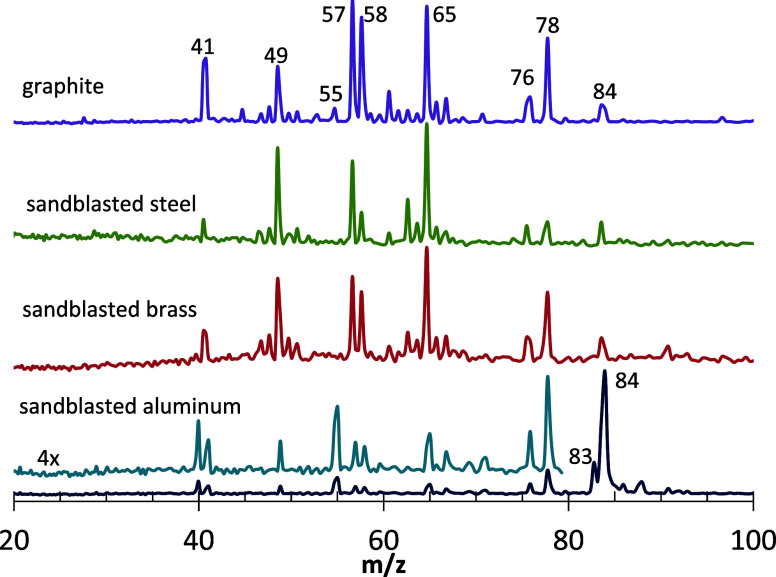
118 nm PI TOFMS at the front edge of gas pulses following 532 nm rod ablation pulses for MDI cross-linked HTPB plus AP on a variety of rod surfaces with varied HTPB:AP ratios. While signals were always stronger on graphite matrices, in each instance, significant new product peaks at *m*/*z* 76 and 78 appeared.

HTPB cross-linking did not add any significant peaks; a similar pyrolytic product composition was observed with and without cross-linking (Figure 2). The cross-linking agent alone, MDI, yielded three major peaks, *m*/*z* 39, 182, and 197; the latter two are significant fragments of MDI along with minor peaks at *m*/*z* 78, 132, 153, 166, 182, 196, 224, 250, and 269 (Figure S1). Several of these peaks (*m*/*z* 78 and 166) could potentially interfere with those of arenes considered in this study. In practice, however, cross-linked HTPB only occasionally displayed small *m*/*z* peaks at *m*/*z* 104 and 197, both apparently due to remnants of the MDI cross-linker.

As shown below, arenes were observed with both neat and cross-linked HTPB in the presence of AP, thus removing the concern about possible interference. Chen and Brill explained a lack of cross-linking influence by showing that the cross-linked MDI decomposes at lower temperatures, prior to HTPB decomposition.^26^ In our experiments there is a limited time scale for chemistry, tens of μs. As a result, any first step time required for breaking the HTPB cross-links could impact the available time for subsequent HTPB pyrolysis chemistry. As for AP, though its thermal decomposition can result in numerous products (e.g., NH_4_Cl, NH_3_, HClO_4_, Cl_2_, N_2_O, NO, O_2_, and H_2_O),^4^ most of them have ionization potentials exceeding 10.5 eV, such that they would not be observed in 118 nm SPI, let alone two photon 266 nm REMPI. In particular, with AP alone on graphite surfaces, no peaks were observed in the arene-relevant range of *m*/*z* > 120 (Figure S2). Since no new HTPB decomposition peaks appeared in the presence of AP compared to its absence, it was assumed that it served primarily as a dehydrogenating agent to form water. The *m*/*z* ranges in Figures 2A and 3 show all of the major peaks. In analogous experiments with neat HTPB+AP, no peaks were observed in *m*/*z* 100–200.

There was only one group of products, specifically of C_6_ size, which showed a striking difference due to the addition of AP. Without AP, the *m*/*z* 83–84 peak, belonging to one or several isomeric hexenes and the corresponding hexenyl radicals (presumably linear due to the small time scale, insufficient for isomerization), was most prominent; a minute *m*/*z* 78 peak was sometimes also evident (Figure 2; Figure 2A, lower scan for neat HTPB). By contrast, for cross-linked HTPB, the *m*/*z* 83–84 signal intensity was reduced with AP addition, now with the appearance of two other peaks, *m*/*z* 78 and 76 (Figure 2). Based on stoichiometric considerations, they must be ascribed to benzene and singlet *o*-benzyne (to be called benzyne henceforth), respectively. The *m*/*z* 76 peak was always accompanied by that of *m*/*z* 78, corroborating their identities: the benzyne in this system must be formed from benzene. Figure 3 shows that these peaks cannot be ascribed to graphite; they were also observed with other rod surface materials.

Because these peaks grow at the expense of the *m*/*z* 83–84 peak, it appears that these two aromatic products are formed by dehydrogenation and cyclization of hexenes. The free radical formed from any hexene can undergo a facile reversible isomerization into the terminal radical, which then undergoes a cyclization; it can then be readily dehydrogenated into benzene,^27^ with a potential further dehydrogenation to benzyne (Scheme 1). A significant part of HTPB double bonds occurs in cis configuration, thus enabling these reactions.^28^ The process becomes irreversible under dehydrogenating conditions once the cyclic free radical intermediate is stabilized by the hydrogen atom release.

**Scheme 1:**
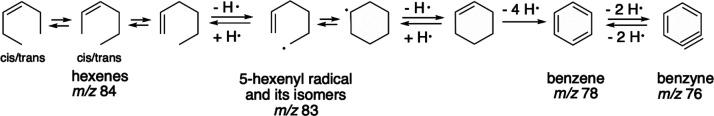
Proposed Pyrolytic Formation of Benzene and Benzyne from Hexenes and the Corresponding Hexenyl Radicals

The formation of an aryne species was not observed for the other aromatic product, toluene, presumably due to its low concentration: Only the *m*/*z* 92 peak, but not 90, was barely seen with 118 nm PI (Figures 2 and 3); it became distinctly discernible only with 266 nm PI (Figures 4–6). Furthermore, the toluene formation was not as enhanced by the AP addition as that of benzene (Figures 2 and 3), apparently because no corresponding acyclic aliphatic C_7_ precursor, with *m*/*z* slightly higher than 92, was formed in sizable amounts. Presumably, these two observations can be linked: the lack (or very low abundance) of other arynes except for benzyne in TOFMS is due to the low amounts of the corresponding precursors. Benzene (but not benzyne) was observed as the major C_6_ species in the other studies conducted at longer time scales.^5,29^ The lack of benzyne observation in those studies is expected due to its instability beyond the microsecond time scale.

**Figure 4:**
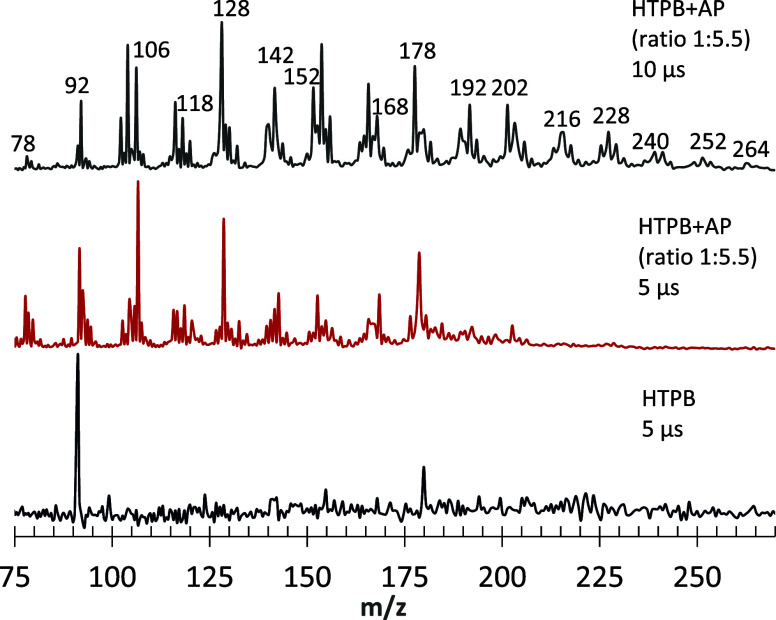
High-MW (*m/* *z* > 110) peaks with and without AP with unfocused 266 nm PI TOFMS following 532 nm rod ablation on graphite slurry of neat HTPB and neat HTPB+AP. PI was carried out with a delay of 5 μs from the front edge of He gas pulses in the first two instances, a point at which association chemistry is enhanced compared to that of the front edge. In the third instance, PI was carried out with a further delay of 10 μs for HTPB+AP. Note the additional association chemistry that was facilitated by this greater delay time. For the top scan, where PAH growth is well developed, Peak spacing within each PAH C_n_ cluster is 2 *m/z*.

**Figure 5:**
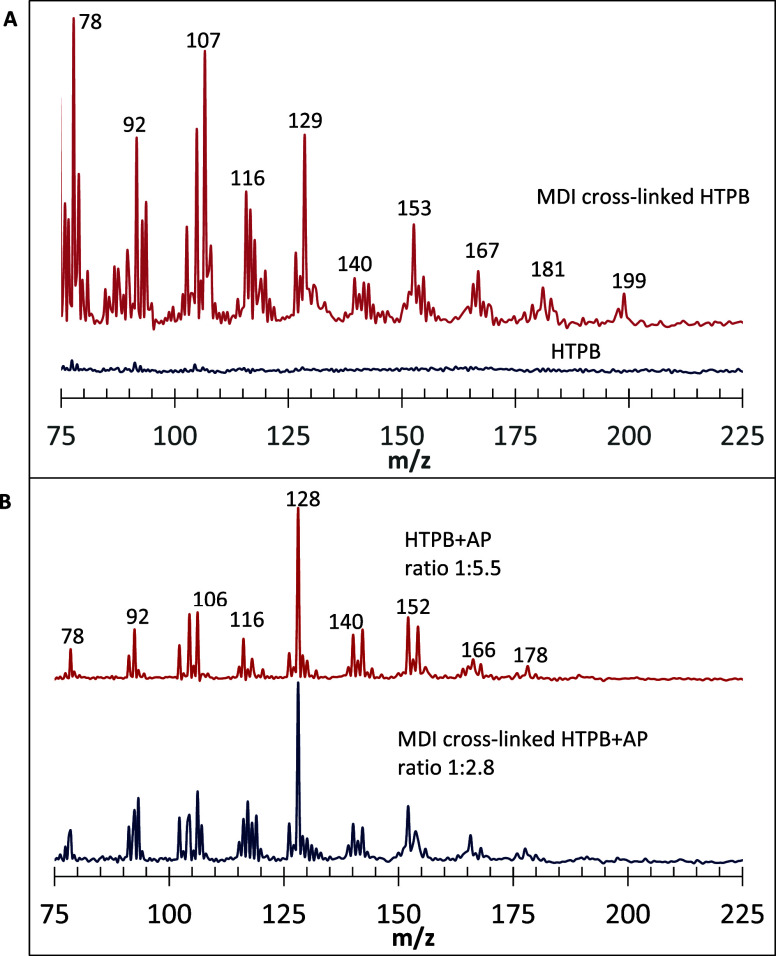
(A) PI using 1 mJ of *focused* 266 nm following 532 nm ablation pulses for MDI cross-linked HTPB and neat HTPB on graphite, taken 2 μs from the front edge of He gas pulses with 3 mJ 532 nm ablation pulses. With unfocused 266 nm PI, the peaks seen here for focused 266 nm *were not detected*. Even with a focused 266 nm, no peaks were observed for neat HTPB. (B) Peaks were observed with 1 mJ *unfocused* 266 nm PI, now at the front edge of gas pulses of He, for HTPB+AP and cross-linked HTPB+AP. Even at the front edge of the gas pulse, with more limited opportunity for high *m*/*z* products to form via association chemistry, PAH product peaks are evident. When the 532 nm pulse energy was increased, the relative intensity of the observed peaks decreased.

**Figure 6:**
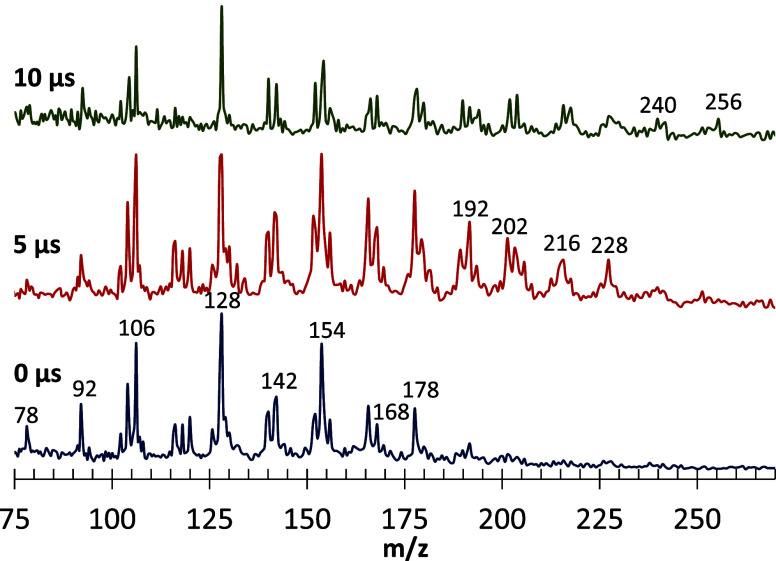
High-MW (*m/z>*100) peaks observed for HTPB in the presence of AP, using unfocused 266 nm PI TOFMS of neat HTPB+AP on brass following 532 nm rod ablation pulses (3.5 mJ) at the front edge (0 μs) of He gas pulses, then 5 and 10 μs from the front edge. The HTPB:AP ratio was 1:2.4.

The addition of AP is pivotal in producing sizable *m*/*z* 76 and 78 peaks, though it is possible that cross-linked HTPB alone may induce MDI cross-linking alone. See Figure 2B, upper scan at 5×, where a minute *m*/*z* 76 peak is evident. The observation of benzyne formation provides significant mechanistic insight. In this context, AP should be viewed as a powerful oxidant significantly affecting the pyrolysis chemistry.

As we have observed, the acyclic C_6_ precursors to benzene appear only at temperatures above 900 °C; benzyne, in turn, can be formed from benzene when the dehydrogenation reactions become dominant. However, the stability of benzyne decreases at temperatures above 800–900 °C, as shown in the seminal paper of Hirsch et al. on benzyne chemistry.^30^ At first sight, this fact would seem to preclude our ability to observe this species. Nonetheless, we can readily observe it: Within ∼10 μs, the initial pyrolysis products, including benzyne, have been cooled in the completed supersonic gas expansions, after which the center stream species, skimmed to form a molecular beam, are in a near-collision free environment. As was the case for our experiments, supersonic expansion MB methods have in numerous past instances been used to stabilize a variety of unstable species for further study.^22,23^ The next step was to determine what products are formed from unstable benzyne when it is not stabilized. This issue is addressed in the next section.

### Detection of Early PAHs by REMPI

In the 118 nm PI TOFMS previously presented, HTPB pyrolysis peaks should roughly scale with species populations. Any possible PAH species above *m*/*z* 100, if present, evidently had small relative populations. To investigate their possible presence, we then used 266 nm two photon REMPI, which should selectively ionize any such PAHs, thus enhancing their peaks.^31^ Indeed, with 266 nm REMPI, a number of new products were observed, as shown in Figures 4–6. These figures have a low *m*/*z* limit at 75, to exclude the low-MW primary pyrolytic product peaks, thus showing only the new aromatic peaks including PAHs (*m*/*z* > 110), the secondary products of association chemical reactions.

We have found that increases in 532 nm pulse energy resulted in a significant diminution of such *m/* *z* > 110 (PAH) peaks in TOFMS (Figure S3). These peaks must have arisen from benzyne; diminished benzyne population at higher pyrolysis temperatures resulted in their diminished production: At higher heating pulse energies, leading to higher pyrolysis temperatures, the benzyne abundance would have decreased. Even when benzyne stabilization is not possible, the case with longer chemistry time scales when it would not be directly observable, its short time scale effect in leading to PAHs would still be present.

Figure 4 shows a significant effect of AP on the appearance of such *m/* *z* > 110 peaks when unfocused 266 nm PI was used. Corresponding results were not observed for neat HTPB (lower scan). The first two TOFMS mass spectra in Figure 4 were taken with a time delay of 5 μs relative to the front edge of the gas pulses, affording a longer chemistry time scale than the front edge. For neat HTPB, the result was only an enhanced *m*/*z* 92 peak with a 266 nm PI (bottom scan). However, no higher aromatics were observed for HTPB without the addition of AP (middle vs bottom scan). With AP addition and an even more extended time for further chemistry, a 10 μs delay relative to the front edge of gas pulses, and PAH peaks at *m*/*z* > 110 were more strongly evident (top scan).

To try to detect any PAH (*m/* *z* > 110) features for neat HTPB, *focused* 266 nm was used (Figure 5A, lower scan). Such a strong fluence could also result in some nonresonant and/or photochemically produced features becoming significant or predominant. However, even under these conditions, there is no evidence of PAH formation. On the other hand, cross-linked HTPB did show evidence of *m/* *z* > 110 peaks (Figure 5A, upper scan), i.e., PAH formation, under these conditions.

On the other hand, the presence of AP resulted in a decisive removal of the difference between neat and cross-linked HTPB, as is seen in Figure 5B. Now the PAH features are evident with unfocused 266 nm. Comparative TOFMS results under the same conditions are shown for both rod portions. Figure 6 shows that the signals assigned to the products of the association reaction are observed on graphite-free metal rods as well. Since no graphite matrix was present, these TOFMS confirm that the aromatic clusters do not arise from extraneous carbon.

In summary, the combination of Figures 4–6 shows that AP is most important in inducing PAH generation, starting with the appearance of *m*/*z* 76 and 78 peaks as their essential precursors. As for the similarities between neat and cross-linked HTPB with AP, shown in Figure 5B, Chen and Brill provided a plausible explanation^26^: The MDI cross-linker *per se* does not influence the HTPB decomposition chemistry; the MDI links decompose and MDI decomposition products vaporize prior to HTPB pyrolysis. In contrast, the first step of neat HTPB pyrolysis, with no cross-links, is exothermic: Stronger bonds are forming than those breaking; i.e., effective polymerization prevails over depolymerization. This first step occurs during the heating ramp, i.e., at relatively lower temperatures. However, as previously noted, HTPB does not start decomposing until the cross-links are gone. As a result, the exothermic step, side reactions, is skipped when cross-linked HTPB is heated quickly, proceeding directly with depolymerization/decomposition reactions. For benzene/benzyne formation, a shorter time scale with cross-linked HTPB may favor highly endothermic dehydrogenation reactions essential for this process. It is also possible that cross-linking increases the system’s microheterogeneity, so some zones favoring dehydrogenation may be formed even without AP. The presence of AP may further increase heterogeneity because this chemical contains both an oxidant (perchlorate) and reducing agent (ammonium cation) and its distribution within HTPB is not even.

Figures 4 and 6 show that the signals corresponding to compounds with *m/* *z* > 110 are more intense when the MB is sampled away from the gas pulse front edge, i.e., with more extended residence time. Consistent with these trends, the upper scan in Figure 4, with a 10 μs delay, shows peaks up to *m*/*z* = 264. Therefore, the observed high MW peaks are due to the products of secondary association chemical reactions. The appearance of the new high-MW peaks is correlated with the appearance of benzene and benzyne; i.e., they are due to extensive dehydrogenation reactions promoted either by high temperature on a short time scale or, particularly, by the presence of AP. When no benzene was formed, e.g., at lower temperatures and no AP, the high-MW peaks did not appear. Notably, as seen in Figure 6, the benzene peak diminishes as the growth of high MW products increases. From the entirety of Figures 2–6, one may conclude that the high-MW compounds form from benzene/benzyne as their essential precursors.

### Stoichiometric Analysis: PAH Growth

The relatively high *m*/*z* peak values (>110) of the ions observed in REMPI-TOFMS (266 nm PI) match the molecular ions of known stable PAHs.^32^ However, tentative assignments are subject to ambiguity in the absence of pure standards or unique fragment ions: The stoichiometric analysis will provide the PAH formulas but cannot distinguish the unsubstituted PAHs from isomers differing in certain ring positions or, sometimes, alkyl derivatives of lower-MW PAHs having the same mass. All PAHs observed were of relatively low MW compared to soot precursors, with only up to 5 rings, accounting for all possible *m*/*z* values of stable PAHs and their methylated derivatives, potentially also ethylated, vinylated, etc.

When using 266 nm REMPI, the observed peak heights and areas should not be viewed as a direct indication of relative species abundance. A more powerful factor determining peak heights is specific compound level absorbances in the vicinity of the set REMPI wavelength, 266 nm, in this work. This value is generally higher for aromatic hydrocarbons compared to alkenes (saturated hydrocarbons do not absorb at this wavelength) and then continues to increase significantly with the number of PAH rings.^33^ Benzyne absorbs predominantly in a range of 160–208 nm;^34^ its absorption near 266 nm and beyond is rather weak because, unlike aromatic benzene with 6 (4n+2) π electrons, benzyne with 8 (4n) π electrons is antiaromatic. As a result, the observed relatively low signal intensity for benzene and benzyne in 266 nm REMPI compared to those of PAHs does not necessarily mean low relative populations of these monocyclic compounds.

In a number of prior publications, benzyne was considered as a source of PAHs including the soot precursor growth in coke during combustion,^35–37^ in interstellar space studies^38^ and in organic synthesis.^39–41^ Hirsch et al. provided a detailed study of benzyne thermal stability and self-reactions at μs time scale.^30^ They showed that benzyne is relatively stable (in an inert atmosphere, with no other hydrocarbons) up to 600 °C, decomposing and forming oligomers at temperatures higher than 800 °C. Given a relatively sizable abundance of the *m/*z 76 ion corresponding to benzyne in our system (Figures 2 and 3), its high conversion rate is expected. If other hydrocarbons are present, benzyne then undergoes multiple reactions, thus becoming a transient species.^37,41^ These reactions are considered below, explaining the formation of the PAHs observed by 266 nm REMPI TOFMS. A remarkable feature of the system studied herein is that the gradual PAH growth may be observed within the 10 μs time scale range, as shown in Figures 4 and 6. The following narrative, referring to Figures 4–6, considers the observed peak clusters, which are 12–14 Da apart, consistent with the PAH growth by one carbon atom per cluster, with or without some hydrogen atoms. The best figure for following the named *m*/*z* values is the top scan of Figure 4 because it includes high-MW PAHs.

### C_8_ Monocyclic Aromatic Product Peaks (not PAHs)

The characteristic molecular ions of both styrene (C_8_H_8_), *m*/*z* 104, and either ethylbenzene or dimethylbenzene (C_8_H_10_, *m*/*z* 106), are relatively abundant. They still yield minor peaks, even smaller in abundance than toluene, *m*/*z* 92, which is a minor product (see Figures 2 and 3 where this peak is small with nonselective SPI). A phenylacetylene peak, *m*/*z* 102, is also visible. These compounds may or may not be produced from benzyne. For example, under dehydrogenation conditions, they can be readily formed by dehydrogenation of 4-vinylcyclohexene, reported as a product of HTPB decomposition in a number of prior studies.^5–9^

### C_9_ Aromatic Product Peaks

The formation of all bicyclic aromatics and several PAHs from benzyne is illustrated in Schemes 2 and 3, compiled according to the literature cited in the text. Of those, Scheme 2 shows the most obvious processes reported in the literature, which are specific to the given molecules, whereas Scheme 3 presents more general alternatives, which are also considered in the literature. The specific reactions are labeled as letters A–G in Scheme 2 and then H–L in Scheme 3. The formation of the simplest bicyclic PAH, indene (C_9_H_8_), *m*/*z* 116 (Figure 4, top scan), was expected. It can be produced from benzyne and propene (or propyl radical) via a so-called ‘aryne ene’ (or ‘Alder ene’) reaction, i.e., the C_3_ fragment attachment to benzyne by the terminal carbon at the C=C bond followed by cyclization, Scheme 2D.^42^ The exact process of indene formation from these reactants has been described in synthetic chemistry.^43^ Minor implied variations, e.g., a similar benzyne reaction with an allyl radical instead of allene, are not shown in Scheme 2. The less pronounced *m*/*z* 118 peak could be due to either the intermediate of the aryne ene reaction or Indane (hydrogenated indene). The peaks of even more hydrogenated species are minor. Hydrogenated species can still be formed in this predominantly dehydrogenating system (in the presence of AP) due to its heterogeneity, as both atomic and molecular hydrogen are abundantly generated in the reactions of benzene/benzyne formation, Scheme 1.

**Scheme 2:**
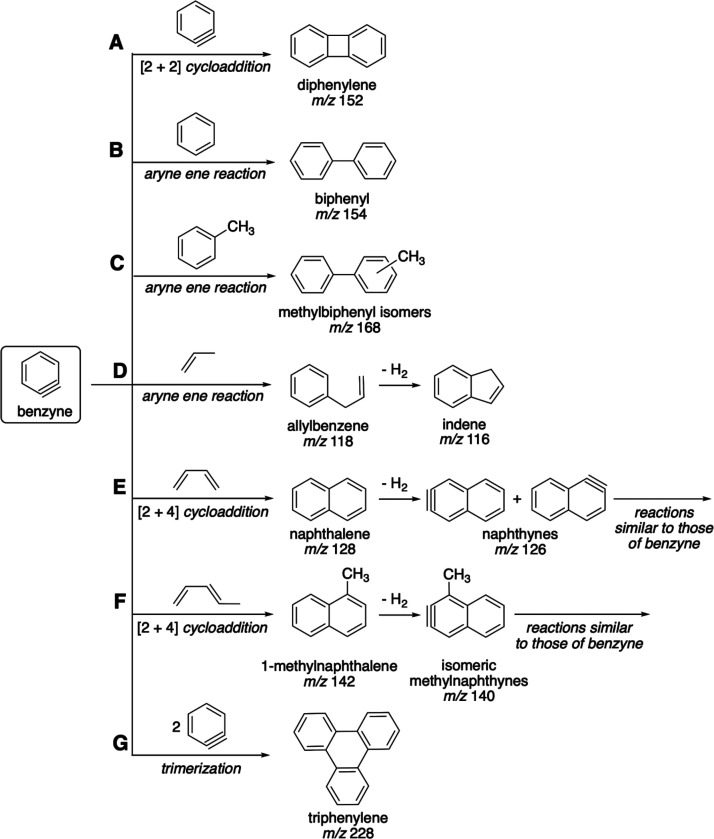
Known Reactions of PAH Formation from Benzyne^a^

a For reactions A–G, see refs 30 and 45,^42^,^42^,^42^ and^43^,^39^,^41^ and^45^, and^30^, respectively.

**Scheme 3:**
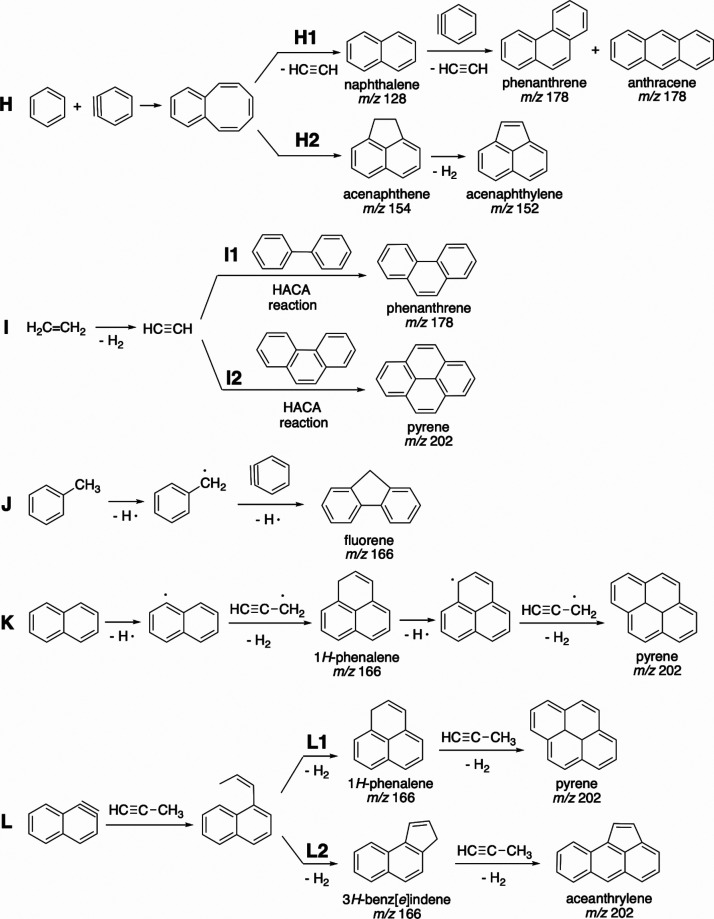
Other Possible Reactions Leading to of Three- and Four-Ring PAHs (refs 40,42,48–50 )

### C_10_ Aromatic Product Peaks

The dominant ion peak, as expected, is that of naphthalene (C_10_H_8_), *m*/*z* 128, accounting for most of the corresponding peak cluster (Figure 4, top scan). Naphthalene is readily formed from benzyne and 1,3-butadiene via the Diels–Alder reaction, also called [2 + 4] cycloaddition^39^ (Scheme 2E). Partially hydrogenated derivatives, *m*/*z* 130 and 132, are present in minor amounts (these signals could also be due to methylated indene and Indane, respectively). A small peak at *m*/*z* 126, presumably of the corresponding aryne derivative (naphthyne), was also detected.

There is one more transformation from benzyne to naphthalene described in the literature. It was discovered as a minor path of the reaction of benzyne with benzene (whose major products are shown in Scheme 3H).^40^ The intermediate, a bicycle with six- and eight-carbon rings, would either isomerize to acenaphthene (Scheme 3H2) or release the molecule of acetylene to generate naphthalene (Scheme 3H1). This path was then observed in the fundamental experimental study conducted by Hirsch et al.^30^ The computational work by Comandini et al. showed that this path significantly enhances the PAH growth in flames whenever benzyne can be formed, with low activation energies for all its steps.^37^ This alternative path of the naphthalene formation illustrates that multiple reactions may occur in this system due to high benzyne reactivity, so only a fraction of possible reactions are shown in Schemes 2 and 3.

Given that the ionization potential of acetylene is 11.4 eV,^44^ above the 10.5 SPI energy of 118 nm photons, we could not directly observe its presence. However, acetylene may be hydrogenated *in situ* to ethylene, which was detected in this system (Figure 2A). Furthermore, according to Hirsch, decomposition to acetylene and diacetylene (1,3-butadiyne) is expected to be the predominant path of benzyne processing at temperatures exceeding 800–1000 °C.^30^ We observed the *m*/*z* 50 peak, perceivably that of the diacetylene molecular ion, and that of its free radical, *m*/*z* 49, using the 118 nm PI (Figures 2A and 3). This observation was possible due to a low diacetylene ionization potential, 10.17 eV.^44^ Ethylene peaks grew in size with the increase of AP:HTPB ratio, i.e., under conditions favoring the benzyne formation (Figure 2A). This observation may be viewed as indirect evidence of benzyne decomposition, with transient acetylene formation. In addition, due to its high reactivity, the acetylene formed may react instantly with several intermediates under dehydrogenating conditions, see Scheme 3I.^45^

### C_11_ Aromatic Product Peaks

The most intense signal is that with *m*/*z* 142, along with 140, plus a minor ion peak at 144 (Figure 4, top scan). The compound with *m*/*z* 142 can be attributed to either methylnaphthalene (C_11_H_10_, two isomers whose formation is shown in Scheme 2F), dimethylindane, or ethylindane (the last two options are combined under the name “C_2_-substituted indane”; this language is used henceforth). The latter option is less likely because the indane ion peak is minor as opposed to indene. As for the *m*/*z* 140 product, this may be an aryne produced from methylnaphthalene, whereas the *m*/*z* 144 signal can be assigned to dihydromethylnaphthalene. The exact reaction of methylnaphthalene formation from benzyne has not been reported, although it can be formed with a C_5_ diene by a Diels–Adler reaction^41,46^ with benzyne, similar to C_4_ dienes, as described in Scheme 2F.

The detection of a compound with *m*/*z* 142 may be viewed as evidence that alkyl-substituted, mostly methyl-substituted PAHs are formed, in addition to unsubstituted ones, because there is no unsubstituted PAH of this mass.^32^

### C_12_ Aromatic Product Peaks

The two major ion peaks are those of *m*/*z* 152 and 154 (Figure 4, top scan) that may be attributed to biphenylene (C_12_H_8_) and biphenyl (C_12_H_10_), respectively. These are two common products of subsequent benzene/benzyne chemistry, although they are formed via different pathways (Scheme 2A and B–C, respectively). The former is a product of benzyne dimerization via a [2 + 2] cycloaddition^47^ (it becomes a major product of benzyne self-reactions at temperatures above 800–900 °C).^30^ The latter can be formed as a result of the aryne ene reaction of benzyne with benzene.^42^ These peaks could also be partially due to acenaphthylene and acenaphthene, respectively (whose formation is illustrated in Scheme 3H2). Henceforth, alkyl- or alkenyl-substituted PAH isomers, e.g., C_2_-substituted naphthalenes, are not mentioned, although their formation is possible. The barely discernible peak at *m*/*z* 150 can belong to either an aryne derivative of biphenylene or ethynylnaphthalene, another product of benzyne self-reactions.^30^

Besides the two-ring PAHs whose formation is explained in Scheme 2, higher-MW PAHs, with a greater number of rings, were observed. Their formation can be similar to the paths shown in Scheme 2, only with two-ring arynes (rather than one-ring, benzyne) as the starting material, as shown for naphthyne in Scheme 3L. Figure 6 presents the evidence of stepwise PAH formation, when smaller-sized PAHs are formed first, followed by larger ones at longer times. Naphthyne and higher-MW arynes have been detected earlier under similar conditions to benzyne.^48^ Several peaks consistent with the *m*/*z* values of such arynes were indeed observed—*m*/*z* 164, 176, 200, 214, and 226. Other possible reactions leading to PAHs with greater numbers of rings are shown in Scheme 3, although the impossibility of predicting all potential reactions in this complex multicomponent system should be noted.

One such pathway (Scheme 3H), 1,4-cycloaddition followed by acetylene abstraction, would enable the PAH growth even without the formation of other arynes except benzyne.^30,37^ This pathway would generate relatively large amounts of acetylene, one equivalent per one PAH formed. However, this combination is not essential as it may be replaced by other reactions depicted in Schemes 2 and 3 leading to PAHs of any possible mass. This system may enable future mechanistic studies of benzyne-driven PAH growth in real time.

### C_13_ Aromatic Product Peaks

The two major peaks observed are those at *m*/*z* 166 and 168 (Figure 4, top scan). The former is likely to be an unsubstituted tricyclic PAH (C_13_H_10_); there are four isomers shown in Scheme 4. Some of them, those having a five-membered ring on a side, may be formed through the reaction of naphthyne with C_3_ products of HTPB pyrolysis, similar to the formation of indene depicted in Scheme 2D (see also Scheme 3L).^42^ An alternate pathway is shown in Scheme 3K. Fluorene is known to be produced from benzyne^30^ by its reaction with benzyl radical (Scheme 3J), which was shown by computation to be barrierless.^43^ The other potential path of fluorene formation could be through the reaction of phenanthrene with methyl or methylene radical.^30^ In the system considered herein, toluene, a potential source of benzyl radicals under dehydrogenating conditions, is known to be formed.^5,6,8,9^ 1*H*-Phenalene can be produced by multiple reactions, e.g., those illustrated in Scheme 3K and Scheme L1.

**Scheme 4:**
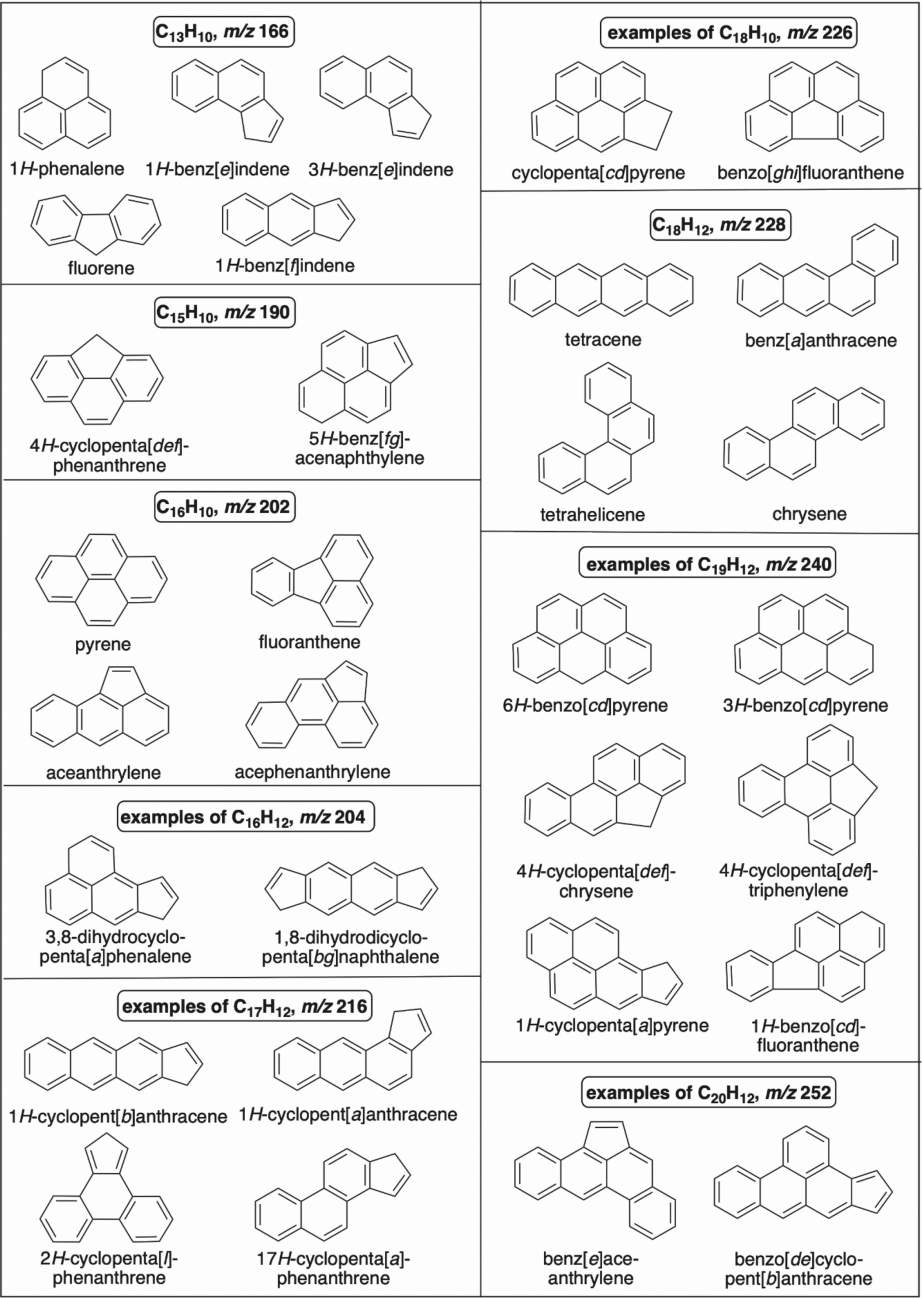
Formulas of the PAHs Mentioned in the Text Except for Those Shown in Scheme 2

### C_14_ Aromatic Product Peaks

The single major ion peak observed at *m*/*z* 178 (C_14_H_10_) (Figure 4, top scan) is definitely due to one of the isomers, phenanthrene and anthracene (or their mixture), well-known products of high-temperature benzyne reactions characteristic for benzene combustion flames.^37^ One way these PAHs can be formed is through the reaction of naphthyne with 1,3-butadiene, similar to the corresponding benzyne reaction shown in Scheme 2E. Several other paths toward the phenanthrene formation are illustrated in Scheme 3H1, I2 and I1. The minor peaks at *m*/*z* 180 and 182 could be partially hydrogenated PAHs.

### C_15_ Aromatic Product Peaks

Two major ion peaks observed at *m*/*z* 190 (C_15_H_10_) and 192 (C_15_H_12_) (Figure 4, top scan) appear to be of different origins. The former must be one of the unsubstituted four-ring PAHs shown in Scheme 4. The latter is most likely due to methylated anthracene or phenanthrene. One of the *m*/*z* 190 PAHs, 5*H*-benz­[*fg*]­acenaphthylene, can be formed from acenaphthylene by reacting with C_3_ products of HTPB pyrolysis, similar to Scheme 3L1, only replacing 1*H*-phenalene with acenaphthylene (we will use the label “type L1 reaction” henceforth).

### C_16_ Aromatic Product Peaks

Some of the peaks of these and larger PAHs can be seen only with residence time extension to 10 μs, see Figure 6, although the major peaks can still be seen in the top scan of Figure 4. The major ion peak observed at *m*/*z* 202 (C_16_H_10_) can be assigned to unsubstituted pyrene or two other four-ring PAH isomers, all shown in Scheme 4. Pyrene can be formed by reacting phenanthrene with acetylene as depicted in Scheme 3I2, so-called hydrogen abstraction-C_2_H_2_ addition, HACA, a common pathway of PAH growth occurring in combustion chemistry^49,50^ when a new ring can be added by C_2_H_2_ addition. A similar known transformation involves the consecutive addition of two propargyl radicals to naphthalene, see Scheme 3K.^51^

The HACA reaction requires prior hydrogen atom abstraction. Thus, the formation of an aryne derivative from its feedstock would enable a similar reaction with a singlet C_3_ fragment, i.e., propyne rather than propargyl radical, path L1 rather than Scheme 3K. A similar modification is possible for Scheme 3I2.

The *m*/*z* 204 peak can be attributed to phenylnaphthalene formed from benzyne and naphthalene according to a type C reaction (Scheme 2C) or to the unsubstituted *m*/*z* 204 PAHs shown in Scheme 4. The latter can be produced from either naphthalene or 1*H*-phenalene by reactions with C_3_ products of HTPB pyrolysis, i.e., type D reaction (Scheme 2D) and L2 reaction (Scheme 3L2).

### C_17_ Aromatic Product Peaks

The single major ion peak observed at *m*/*z* 216 (C_17_H_12_) (Figure 6 and the top scan of Figure 4) could be due to unsubstituted PAHs, the examples of which are shown in Scheme 4. From this point on, the number of potential isomers for a given *m*/*z* value becomes rather large, so only the representative PAHs are included in Scheme 4. The same can be said about the reactions leading to their formation, which may be envisioned by extrapolating the reactions shown in Schemes 2 and 3 from benzyne and naphthyne to their multiring analogs. For example, the aryne derivatives of anthracene and phenanthrene can react with C_3_ products of HTPB decomposition similar to the corresponding reaction of naphthyne, Scheme 3L2.

### C_18_ Aromatic Product Peaks

The single major ion peak observed at *m*/*z* 228 (C_18_H_12_) (Figure 6 and the top scan of Figure 4) is most likely due to a mixture of unsubstituted nonfused PAHs with four six-membered rings, e.g., tetracene, chrysene, benzanthracene (shown in Scheme 4) and triphenylene, whose potential formation is illustrated in Scheme 2G.^30^ There is a smaller peak of the corresponding partially hydrogenated hydrocarbon, *m*/*z* 230, although this *m*/*z* value is also consistent with that of terphenyl, which could be formed by a two-step reaction of benzyne with benzene similar to the biphenyl synthesis, see the type B reaction, Scheme 2B. The *m*/*z* 226 peak could be due to the other group of unsubstituted PAHs shown in Scheme 4.

### Higher MW Aromatic Product Peaks

The observed *m*/*z* 240 peak (Figure 6 and the top scan of Figure 4) may be due to either unsubstituted PAHs, examples of which are shown in Scheme 4, or methylated derivatives of PAHs with *m*/*z* 226. The other peak of this C_19_ cluster, *m*/*z* 242, most likely belongs to methylated PAHs with *m*/*z* 228. This was the last well-defined peak cluster with large peaks. In some experiments, smaller peaks at *m*/*z* 252 and 254 (C_20_ PAHs, some of them are exhibited in Scheme 4) were detected. Most of them can be produced by the extension of the reactions shown in Schemes 2 and 3 to multiring PAHs as feedstocks. Other reactions are possible; for example, some PAH growth may involve reactions of their free radical derivatives rather than arynes, yet similar to those considered above.^52^ Aryne-induced transformations, which commence with hydrogen elimination, are similar not only to HACA (type I reactions, Scheme 3I) but also to PAC (phenyl radical addition/cyclization)^53^ pathways accounting for the PAH growth in flames. HACA may produce not only six-membered, but also five-membered rings in fused PAH structures.^54^ Other potential reactions may involve methyl^55^ or methylene^49^ radicals. However, these species are less likely to be generated in abundance below 1500–2000 °C whereas, as shown above, benzyne and higher-MW arynes are present in substantial concentrations at lower temperatures considered in this study.

### Extended Growth Observed by EGA-MS

The experiments discussed above could not answer one question, namely, whether PAHs can be formed with neat HTPB in the absence of AP on a longer chemistry time scale. To address this issue, EGA-MS experiments were conducted using varied temperature ramps. In the experiments with a ramp from 100 to 1000 °C, virtually no peaks were observed having the *m*/*z* values matching those of PAHs (Figures S4a,b). The experiments with a ramp from 500 to 1000 °C yielded similar results (Figure S4c). Apparently, the massive evolution of gas phase pyrolysis products occurring at temperatures below and near 500 °C in the absence of AP, when benzyne formation is hindered,^30^ resulted in mere evaporation of potential PAH precursors, without their reactions with benzyne. As a result, the next steps of the temperature ramp occurred after the removal of reactive intermediates, thus, yielding no PAH products. Therefore, the rest of the experiments were performed isothermally, when the sample was introduced into the EGA-MS pyrolyzer preheated to a desired temperature, and then the data acquisition was conducted continuously for 10 min. The detailed peak assessment was performed at 15 s (the earliest possible time due to instrument limitations), 24, 54, and 96 s.

Only the results obtained at 15 s at 700 and 800 °C are presented in Figure 7; the rest of the scans for these temperatures are shown in Figure S5c,d. A wide range of peaks was observed, apparently reflecting the greater impact of association chemical reactions as opposed to shorter time scales (see the TOFMS with nonselective PI, Figures 2 and 3). A number of ions associated with PAHs were detected, listed in Table 1 for 15 s samples. However, at lower temperatures, 500 and 600 °C, the major ions detected had odd *m*/*z* values (Figure S5a,b), so they were not PAHs. This observation is consistent with low benzyne stability at those temperatures.

**Figure 7:**
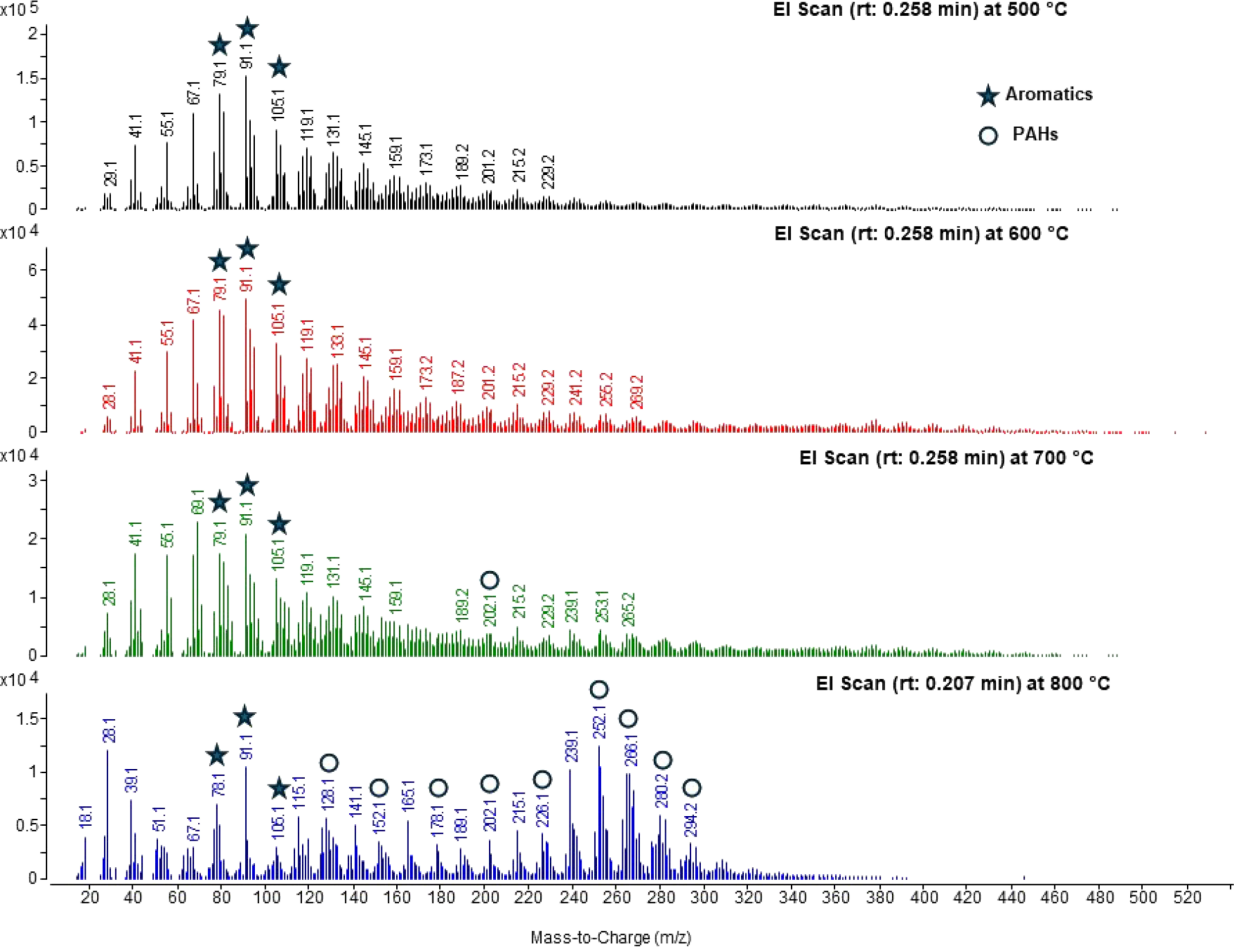
EGA-MS mass spectra obtained in isothermal pyrolytic experiments at 15 s with the temperatures 500, 600, 700, and 800 °C. The stars denote monoaromatic species, and the circles are representative of PAHs.

**Table 1:** Occurrence of Compounds Including PAHs and Aromatic Hydrocarbons in the MS Spectra Obtained (1) by Using EGA-MS following the *In Situ* HTPB Pyrolysis at Varied Pyrolytic Temperatures for 15 s of Residence Time and (2) by Pyr-GC-MS (See Figure 8 for Chromatograms; the Standards’ Chromatograms Are Shown in Figure S7a and Their MS Spectra – in Figure S7b)­^f^

	**EGA-MS analysis at isothermal temperatures and relative abundance of ions**					**TD-Pyr-GC-MS analysis for identification of compounds.**			
formula	MW	500 °C	600 °C	700 °C	800 °C	*t*_R_, min	peak #	compound detected	Confirmation T(%) or S match^a^
C_4_H_6_	54	++++	++++	++++	++++	4.09	1	butadiene	T (95)
C_5_H_6_	80	++++	++++	++++	++++	4.46	2	cyclopentadiene	T (80)
C_6_H_8_	66	++++	++++	++++	++	5.17	3	cyclohexadiene	T (95)
C_6_H_6_	78	++	++	+++	++++	5.19	4	benzene	T (98)
C_7_H_8_	91^b^	++++++	++++++	+++++	++++	6.05	5	toluene	T (98)
						6.88	6	ethylbenzene	T (98)
C_8_H_10_	106	+++	+++	+++	++	6.97	7	xylene	T (99)
C_8_H_8_	104	+	+	++	++	7.16	8	styrene	T (92)
C_9_H_10_	118	++	++	++	++	8.04	9a	propenylbenzene	T (96)
C_9_H_8_	116	++	+	+	++	8.50	9b	indene	T (96)
C_10_H_10_	130	++	++	++	+++	9.90	10	methylidene	T (98)
C_10_H_8_	128	+++	+++	+++++	++++	9.64	11	naphthalene	S
C_11_H_10_	142	++	++	++	+++	10.39	12	2-methylnaphthalene	S
						10.50	13	1-methylnaphthalene	S
C_11_H_12_	144	++	++	++	++			ND^c^	
C_12_H_8_	152	++	+	++	+++	11.40	14	biphenylene^d^	T (97)
							14	acenaphthylene	S
C_12_H_10_	154					11.61	15	acenaphthene	S
C_12_H_12_	154	+	+	++	++	10.89	13a	ethenylnapthalene	T (92)
C_13_H_12_	166	+	+	++	++	12.20	16	fluorene	S
C_14_H_10_	178	++	++	++	+++	13.30	17a	phenanthrene	S
						13.34	17b	anthracene	S
C_14_H_12_	180	+	+	+	++	12.85	16b	methylfluorene	T (83)
C_15_H_10_	190	+	+	+	++			ND^c^	
C_15_H_12_	192	+	+	+	++	13.85	18a	methylphenanthrene ^e^	T (81)
						14.97	18b	methylanthracene ^e^	T (85)
C_15_H_14_	194	+	+	+	+			dihydromethylanthracene	T (79)
C_16_H_10_	202	++	++	++	+++	14.67	19	fluoranthene	S
						14.93	20	pyrene	S
C_16_H_12_	204	+	+	+	++	14.17	18c	phenylnaphthalene^e^	T (79)
C_16_H_14_	206	+	+	+	+	15.10		dimethyl anthracene^e^	T (81)
								tetrahydropyrene^e^	T (81)
C_17_H_12_	216	+	++	++	++	15.91	19a	methylfluoranthene^e^	T (78)
						15.99	20a	methylpyrene^e^	T (70)
C_18_H_12_	228	+	++	++	+++	16.35	22	chrysene	S
						16.30	21	benz[*a*]anthracene	S
C_18_H_14_	230	+	+	+	++			ND^c^	
C_19_H_14_	242	+	+	+	+++	16.84	22a	methylbenz[*c*]phenanthrene^e^	T (74)
								methylchrysene^e^	T (73)
								methylbenz[*a*]anthracene^e^	T (72)
C_20_H_16_	240	+	+	++	++++	17.05	22b	dimethylbenz[*a*]anthracene	T (70)
C_20_H_10_	250	+	+	+	+++			ND^c^	
C_20_H_12_	252	+	+	++	++++++	17.86	23	benzo[*b*]fluoranthene	S
							23b	benzo[*k*]fluoranthene	S
						18.27	24	perylene	T (70)
						18.35	24	benzo[*a*]pyrene^e^	S
C_20_H_14_	254	+	+	++	+++++			ND^c^	
C_21_H_14_	266	+	+	++	+++++	18.76	25	methylbenz[*j*]aceanthrylene^e^	T (76)
								dibenzo[*a,h*]fluorene^e^	T (77)
C_22_H_12_	276	+	+	+	+++			ND^c^	
C_22_H_14_	278	+	+	+	+++			ND^c^	

a Confirmation of the MS identification: T denotes “tentative,” based on the MS library % match, S denotes the confirmation by the corresponding chromatographic standard, by matching its retention time.

b Tropylium ion, an essential fragment of any monoalkylsubstituted benzene.

c ND denotes “not detected” (by Pyr-GC-MS as opposed to EGA-MS).

d The peak of a PAH with *m*/*z* 152 matches the retention time of acenaphthylene. Nonetheless, the MS spectra match is closer to biphenylene showing the ethylene fragment, which is not characteristic of acenaphthylene. These two PAHs most likely coelute.

e Several isomers showed a similar match with NIST MS spectra for these peaks. Since no chromatographic standards were available, they all are listed.

f The + to + +++++ symbols indicate the ions’ relative abundance in EGA-MS normalized to the maximum *m*/*z* ion peak area observed (“base peak” in MS terminology)

The characteristic ions used as well as their time profiles are shown in Figure S6. Low-MW aromatics, particularly toluene along with naphthalene, dominated the low temperature mass spectra obtained by EGA-MS below 700 °C (Figure S6a,b). The observed low benzene concentration is consistent with the proposed mechanism of PAH growth induced by the formation of benzyne, for which benzene, in turn, is the essential feedstock. At higher temperatures, 700 and 800 °C, aromatic products started to dominate, i.e., PAHs along with monoaromatic hydrocarbons (Figure S6c,d, respectively). The same trend can be seen in Table 1: higher-MW PAHs were formed at the expense of low-MW aromatics toward the highest temperature used. These observations indicate the stepwise PAH formation, with low-MW aromatics serving as building blocks of higher-MW PAHs.

The scans in Figure S6c,d are shown with the subtraction of the previous scan. As one can see, no new PAH ions were detected at the times longer than 15 s, thus setting the upper bound of the PAH formation time scale. The actual time scale of PAH formation is further lowered when matching the maximum MW values observed in TOFMS and the EGA-MS experiments.

They matched indeed, showing the PAH formation only up to about *m*/*z* 250–280 (Table 1 vs Figures 4–6). Thus, the process is evidently complete within tens of microseconds characteristic for MB-TOFMS. This finding is consistent with benzyne being the main source of PAHs regardless of the time scale. Apparently, higher-MW, multiring arynes are expected to be less reactive than benzyne, and so, the PAH growth is limited. In addition, the TOFMS obtained indicate that these high-MW arynes are present in much smaller amounts.

The use of EGA-MS has two significant advantages over Pyr-GC-MS due to the absence of a chromatographic column. First, all of the detectable products are detected within the selected MS *m*/*z* value range, regardless of their MW, polarity or any other factor affecting column elutability. The second advantage, specific for isothermal experiments conducted in this study, is the possibility of obtaining the product profiles at a relatively short residence time, below 1 min.

### Pyrolytic Product Identification via Pyr-GC-MS

Pyr-GC-MS requires longer heating times, but unlike EGA-MS, it enables product identification using the MS EI library, based on specific ion fragmentation patterns as well as retention time comparison with standards. Based on the EGA-MS results, Pyr-GC-MS analysis were performed on the samples at three isothermal temperatures, 700, 800, and 900 °C. The chromatograms, with the pertinent peaks marked with numbers, are shown in Figure 8. The PAHs identified are listed in Table 1. As shown under Materials and Methods, the PAH identification was conducted by both MS and matching the analyte retention time with that of the corresponding pure standard (whose chromatograms and mass spectra are shown in Figure S7a,b). The GC temperature program was sufficient to clearly separate and identify all of the observed PAHs, except for chrysene, for which a slightly different temperature gradient had to be applied (Figure S8). Additional PAH structures identified by Pyr-GC-MS are shown in Figure S9.

**Figure 8:**
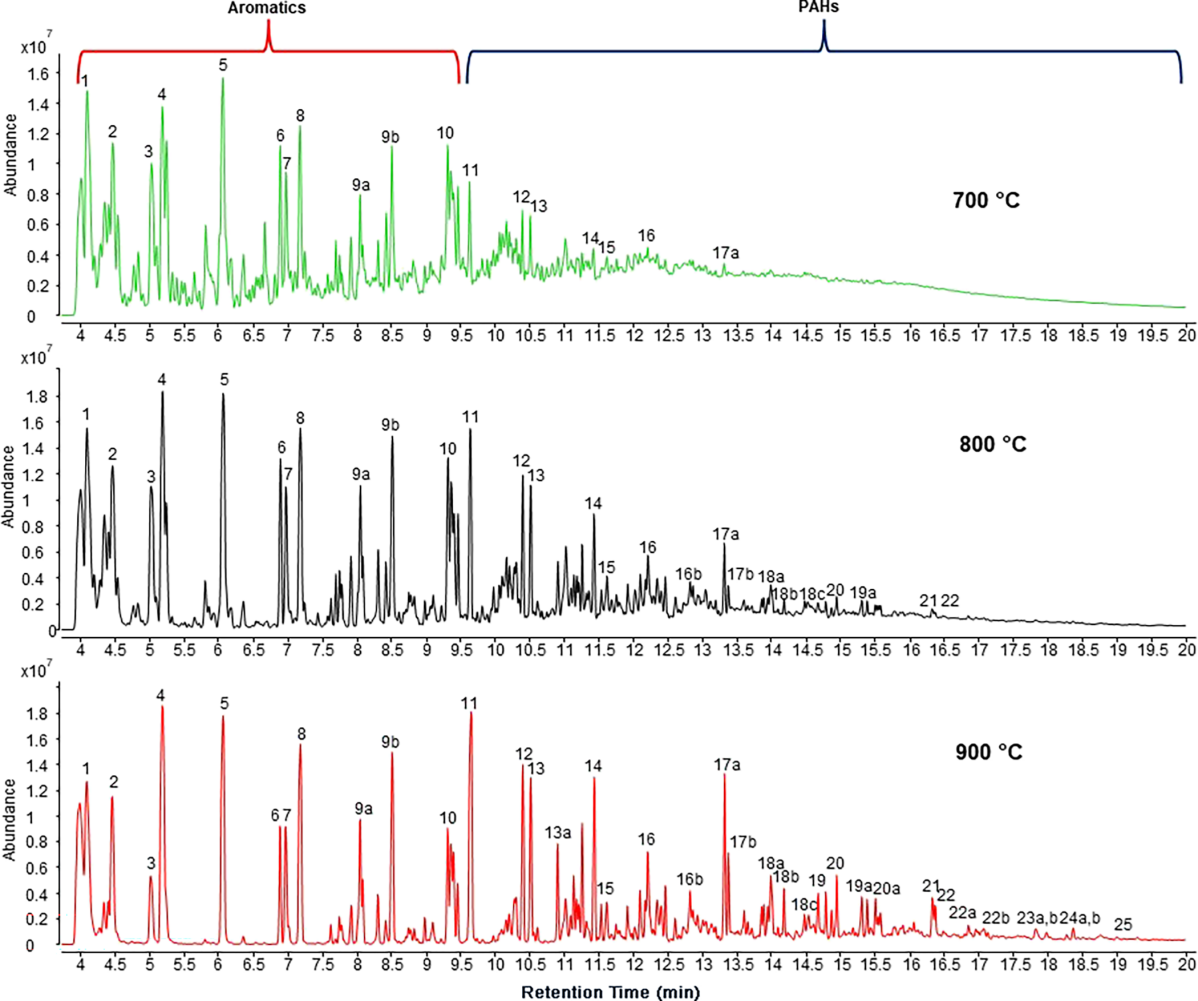
Pyr-GC chromatograms obtained at 700, 800, and 900 °C. The peak labels correspond to those shown in Table 1 in the column shadowed in gray.

The Pyr-GC-MS results corroborated several conclusions based on TOFMS and EGA-MS. The highest-MW identifiable PAHs were those of 276 Da; the PAHs of higher MW did not yield discernible peaks. The agreement obtained on PAH size between all three methods used entails similar time scales for PAH growth, i.e., the bulk of the PAH formation is complete within just tens of microseconds. The major nonaromatic products observed by Pyr-GC-MS were identified as 1,3-butadiene, cyclopentadiene and cyclohexadiene. The first two were the major C_4_ and C_5_ products identified in TOFMS experiments. Cyclohexadiene is an essential intermediate of benzene/benzyne formation from its aliphatic precursors (Scheme 1). Benzene was among the highest peaks, too, confirming its significance as one of the major products and a presumed PAH precursor.

Corroborating the EGA-MS results, toluene yielded one of the most pronounced peaks and the largest one among those produced by aromatic products, followed by ethylbenzene and *o-*xylene. Apparently, the monocyclic products of sizes larger than C_6_ become more prominent at longer residence times than those observed in TOFMS. Several studies conducted at longer residence times corroborate this assumption by indicating 4-vinylcyclohexene as one of the major products,^5–9^ whereas we did not observe it in TOFMS at short time scales at all.

The PAH profile obtained by Pyr-GC-MS shows several partially hydrogenated PAHs, corroborating the TOFMS results and the assumption that both hydrogenation and dehydrogenation processes occur in this system. There are also several alkylated PAHs, mostly methylated. It is of note that the MS detection in EGA-MS could not identify either alkylated or hydrogenated PAHs; their masses were not as specific as those of unsubstituted fully conjugated PAHs. The observed diversity of methyl PAHs, combined with the high abundance of toluene shown by both EGA-MS and Pyr-GC-MS, may indicate a significant role of toluene in PAH growth, even though toluene was observed merely in minor amounts in TOFMS experiments. As noted in the previous paragraph, toluene becomes a major product of HTPB pyrolysis on a longer time scale, just as observed earlier by Rao and Radhakrishnan, and Chiaverini et al.^5,9^

Most of the PAHs detected by EGA-MS were then observed by Pyr-GC-MS, except for *m*/*z* 190, 226, 240, 250 and higher than 276. Some PAHs, particularly those of higher MW, could either be formed in amounts too low to be reliably identified by GC-MS, or “lost” within the chromatographic baseline. Furthermore, the absence of some PAH ions can mean that they are intermediates of higher-MW PAH formation. Given this limitation, the observation of only specific PAH isomers and homologues combined with the virtual lack of the others should be noted. Namely, among the isomers with *m*/*z* 166 (Scheme 4), only fluorene was found, along with several of its methylated and hydrogenated derivatives, thus, indicating that this PAH is formed selectively compared to its isomers. The same can be said about pyrene and fluoranthene formation, which were observed by Pyr-GC-MS, unlike their isomers (Scheme 4).

As a result, the list of PAH isomers formed should be narrowed as well as the list of reactions of PAH formation from benzyne. The adjusted network of transformations consistent with the Pyr-GC data is shown in Scheme 5, with the exception of benzyne self-reactions, e.g., that yielding biphenylene. The analysis of the observed products indicates that the only PAH structure with a five-membered ring on the side of the aromatic core is indene. Also, the only structure with four fused rings is that of pyrene and its higher-MW derivatives. The lack of the products with *m*/*z* 190 and fused rings, one of which is five-membered, illustrates this trend. This observation can be explained by having two key two-ring PAH intermediates, naphthalene and indene, each of them producing specific products.

**Scheme 5:**
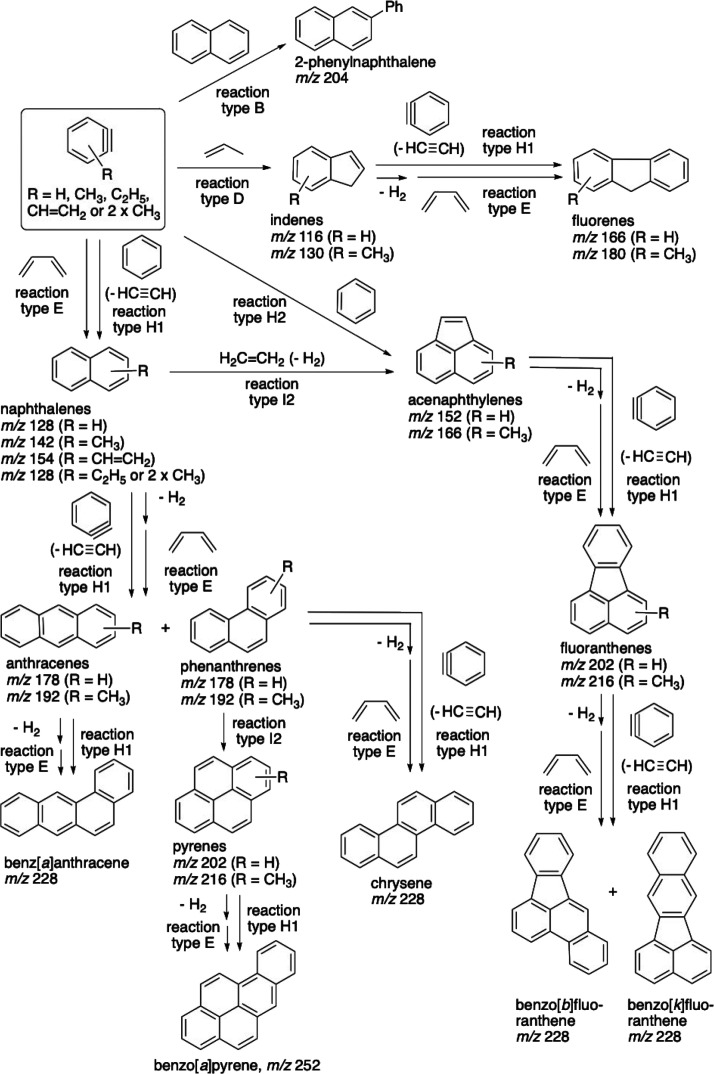
Most Probable Reaction Sequences Consistent with TOFMS, EGA-MS, and Pyr-GC-MS Results, Except for Benzyne Self-Reactions^a^

a The “-H_2_” reactions are those forming the corresponding aryne intermediates.

Furthermore, only benzene and naphthalene featured multiple alkylated derivatives. This feature may indicate that only these two intermediates form reactive arynes in sufficient amounts. If other arynes, i.e., derivatives of indene, anthracene, phenanthrene, acenaphthylene, and pyrene, are not formed in significant amounts, then the corresponding reactions affording aryne structures (marked with “-H_2_” in Scheme 5) should be considered less important than the competing HACA paths. In turn, this inability to form reactive arynes beyond naphthyne may explain the observed lack (the absence of) of PAHs with six and more rings.

This study does not aim at finding a complete set of reactions resulting in PAH growth with benzyne as the feedstock. This can be done in subsequent studies using this system for the efficient benzyne generation. It is of note that not only biphenylene, the product of benzyne self-reaction reported earlier,^30^ was observed in this study. In addition, a number of other PAHs were detected, evidencing the benzyne reactions with other HTPB pyrolytic products of C_2_–C_7_ size as shown in Schemes 2 and 3. The observation of a relatively large number of substituted naphthalenes, including those with C=C bonds in lateral chains, may indicate a significant role of these intermediate PAHs in the further PAH growth.

## Conclusions

The formation of PAHs as a result of HTPB pyrolysis was observed. It occurs on a longer time scale in oxidant-free neat HTPB pyrolysis, but either the HTPB cross-linking or the addition of an oxidant, AP, shortens the time scale to microseconds. The role of AP is the facilitation of oxidation, i.e., dehydrogenation reactions, including the key pathways leading to the production of benzyne and similar “aryne” intermediates of larger size PAH formation. Once such a key PAH precursor is formed, it stabilizes via its addition reaction with a suitable aliphatic or aromatic product of HTPB pyrolysis to enable the PAH growth. The observed products may be accounted for by unsubstituted PAHs up to MW > 250–260 Da plus their alkylated (mostly methylated) derivatives and partial hydrogenation products. The process time scale appears to finish within tens of μs, given that PAHs of similar size were detected by short time scale TOFMS and much longer time scale EGA-MS and Pyr-GC-MS. This system may serve for future studies of benzyne-driven PAH growth. Given that PAHs were observed by Pyr-GC-MS in large amounts, being almost the only significant GC-elutable products of HTPB pyrolysis, the environmental impact of burning solid composite propellants may need serious consideration.

## Supplementary Material



## Notes

118 nm PI TOFMS of MDI as well as AP, unfocused 266 nm PI TOFMS of HTPB+AP, EGA-MS thermograms and the corresponding MS scans, EGA-MS thermograms of neat HTPB pyrolysis and the corresponding MS scans, using both temperature ramps and isothermal regimes with total and extracted ionic currents, GC-MS chromatograms of PAH standards as well as their peak identification, MS spectra of PAH standards, application of standard addition method and optimized temperature ramp rate for chrysene identification, chemical structures of compounds identified by Pyr-GC-MS (PDF)
