# Supplementary material for: PAH Induction upon Pyrolysis of Hydroxyl-Terminated Polybutadiene-Based Solid Rocket Fuels
Source: J Phys Chem A. 2025 Jul 7;129(28):6356–73. doi: 10.1021/acs.jpca.5c03340 (PMC12278256; doi:10.1021/acs.jpca.5c03340)
Supplement: Supplementary file 1 [file jp5c03340_si_001.pdf]

PAH Induction upon Pyrolysis of Hydroxyl-Terminated Polybutadiene Based Solid Rocket Fuels

Valeriia Karpovych[1], Nataliia Haiduk[1], Eugene Oga[2], Nafisa Bala[2], Alena Kubátová[2], Evguenii Kozliak[2*], Mark Sulkes[1]*

[1] Tulane University, Department of Chemistry, New Orleans, Louisiana 70118, United States

[2] University of North Dakota, Department of Chemistry, Grand Forks, North Dakota 58202, United States

*Address correspondence to Mark Sulkes, cm06acf@tulane.edu, or Evguenii Kozliak, evguenii.kozliak@und.edu

Supporting Figures

Figure S1. 118 nm PI TOFMS of the cross-linking agent, MDI.

Figure S2. 118 nm PI TOFMS of ammonium perchlorate (AP).

Figure S3. Unfocused 266 nm PI TOFMS of HTPB+AP.

Figure S4a. EGA-MS thermograms and the corresponding MS scans obtained by HTPB heating from 100 to 900 °C with a gradient of 100 °C/min.

Figure S4b. EGA-MS thermograms and the corresponding MS scans obtained by HTPB heating from 100 to 900 °C with a gradient of 100 °C/min.

Figure S4c. EGA-MS thermograms and the corresponding MS scans obtained by HTPB heating from 500 to 1,000 °C with a gradient of 50 °C/min.

Figure S5a. EGA-MS thermograms and the corresponding MS scans using TIC (total ion current) showing no detectable presence of PAH ions at 500 °C.

Figure S5b. EGA-MS thermograms and the corresponding MS scans using TIC (total ion current) showing no detectable presence of PAH ions at 600 °C.

Figure S5c. EGA-MS thermograms and the corresponding MS scans using TIC (total ion current) showing trace presence of PAH ions at 700 °C.

Figure S5d. EGA-MS thermograms and the corresponding MS scans using TIC (total ion current) showing the presence of PAH ions at 800 °C.

Figure S6a&b. EGA-MS thermograms and the corresponding MS scans using EIC (extracted ion current) showing a possible trace presence of PAH ions at 500 °C and 600 °C.

Figure S6c&d. EGA-MS thermograms and the corresponding MS scans EIC (extracted ion current) showing a possible presence of PAH ions at 700 °C and 800 °C.

Figure S7a. GC-MS chromatograms of PAH standards used for the identification of peaks shown in Table 1.

Figure S7b. MS spectra of PAH standards.

Figure S8. Application of standard addition method and optimized temperature ramp rate for chrysene identification.

Figure S9. Chemical structures of compounds identified by Pyr-GC-MS.

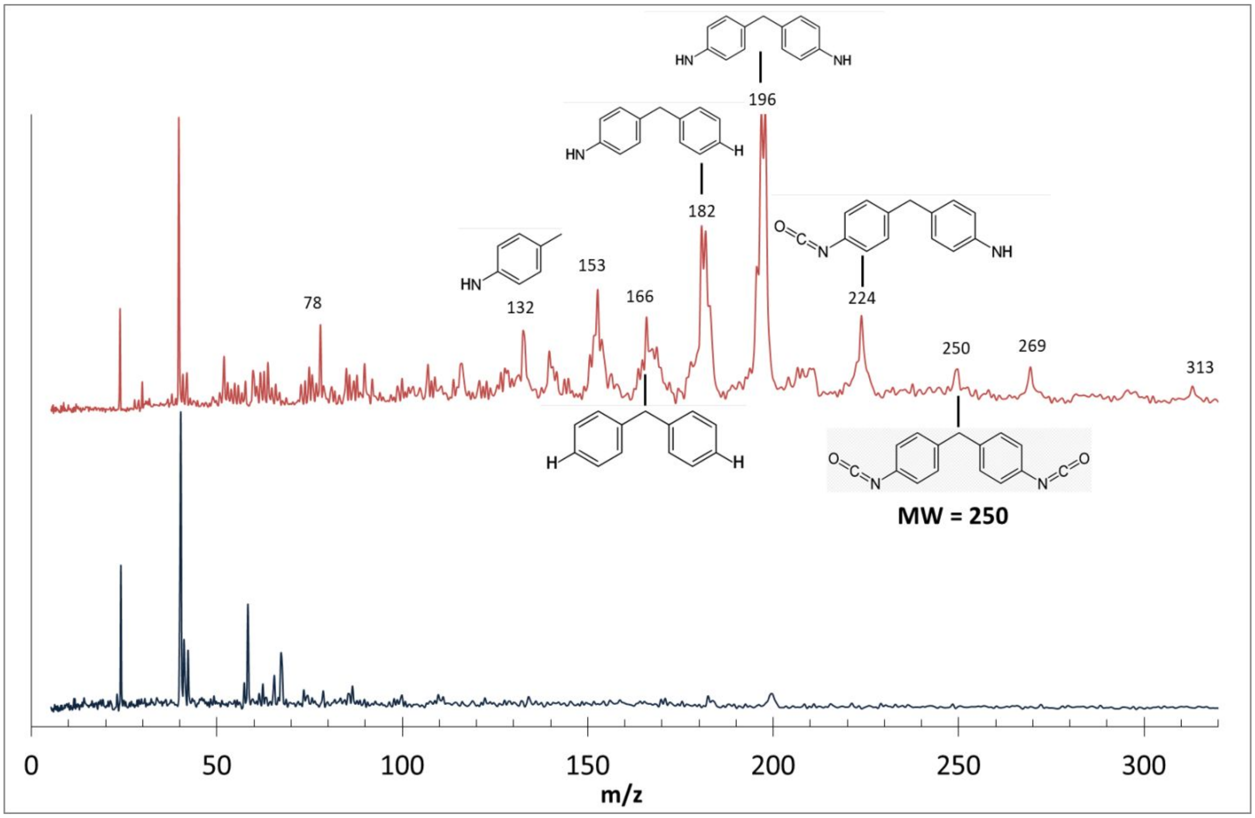

Figure S1. 118 nm PI TOFMS of the cross-linking agent, MDI. The lower scan is near the front edge of the gas pulses; the upper scan is a sum of scans with a mean downstream delay of 10 µs. Further downstream, the pyrolysis temperatures are lower, as is evidenced by the presence of a small MDI parent peak (*m/z* 250) as well as some relatively large fragments with associated structures shown. There is more time for association chemistry further downstream. As a result of such association, some peaks with m/z values above the parent are evident. The MDI was deposited over a graphite deposition on a ceramic rod. The 532 nm pulse energy was 3 mJ. He carrier gas was employed.

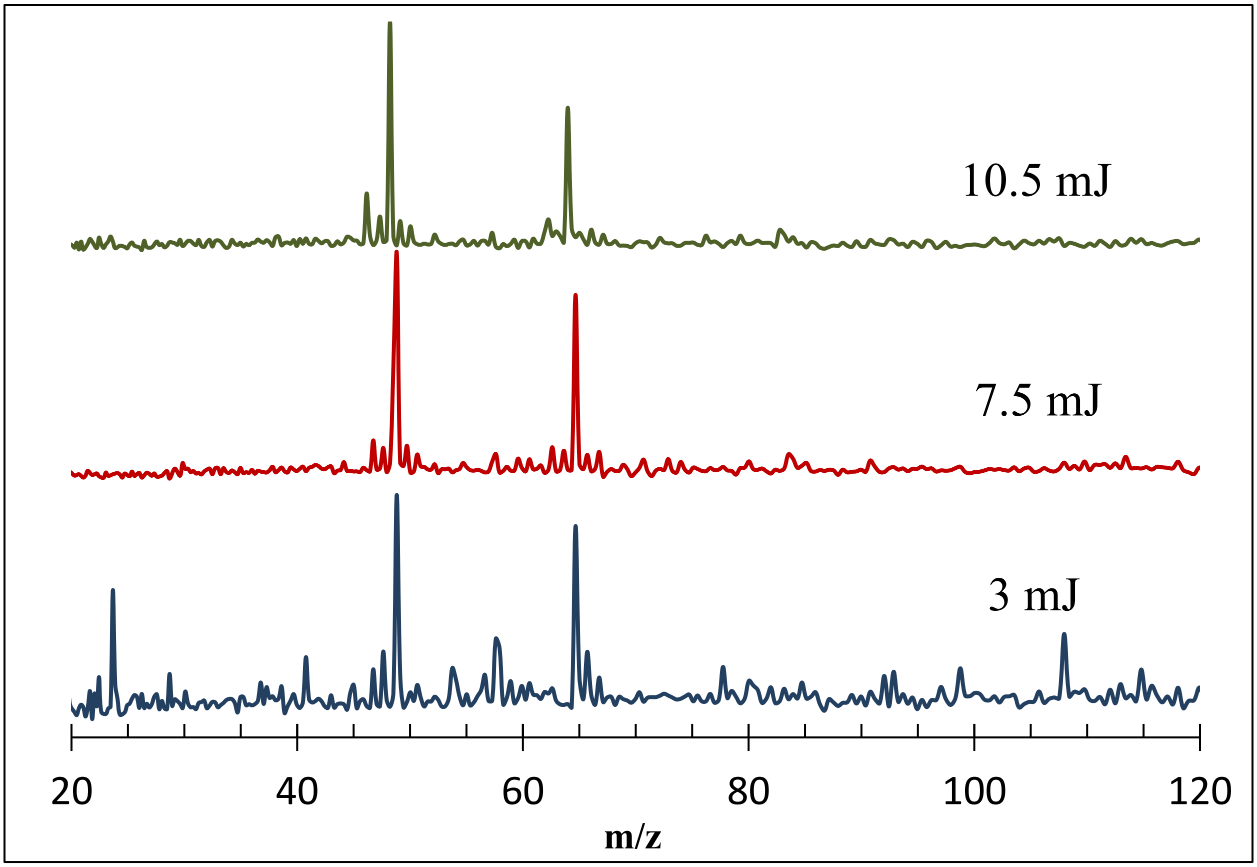

Figure S2. 118 nm PI TOFMS of ammonium perchlorate (AP). Excitation was at the front edge of He gas pulses. The AP deposition was on graphite deposition on a ceramic rod. The 532 nm pulse energies are shown. These peaks appear to derive from the graphite layer. Signals were very weak relative to the detector voltage normally used for HTPB product peaks. In no instances were any peaks discernible above m/z 120.

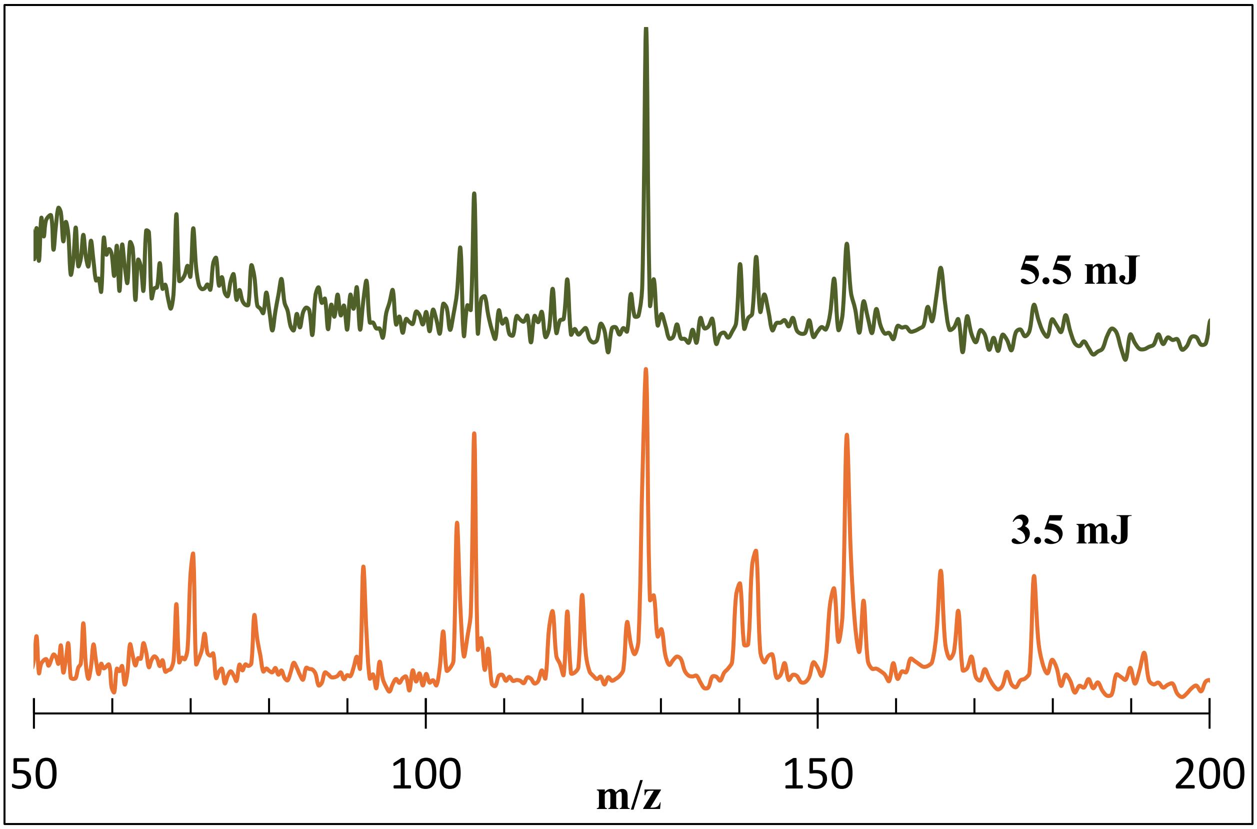

Figure S3. Unfocused 266 nm PI TOFMS of HTPB+AP. Excitation was at the front edge of He gas pulses. The 532 nm pulse energy is shown in both cases. Mean depositions on a brass rod were of HTPB (2.7 mg/cm2) followed by AP (6.4 mg/cm2) on brass.

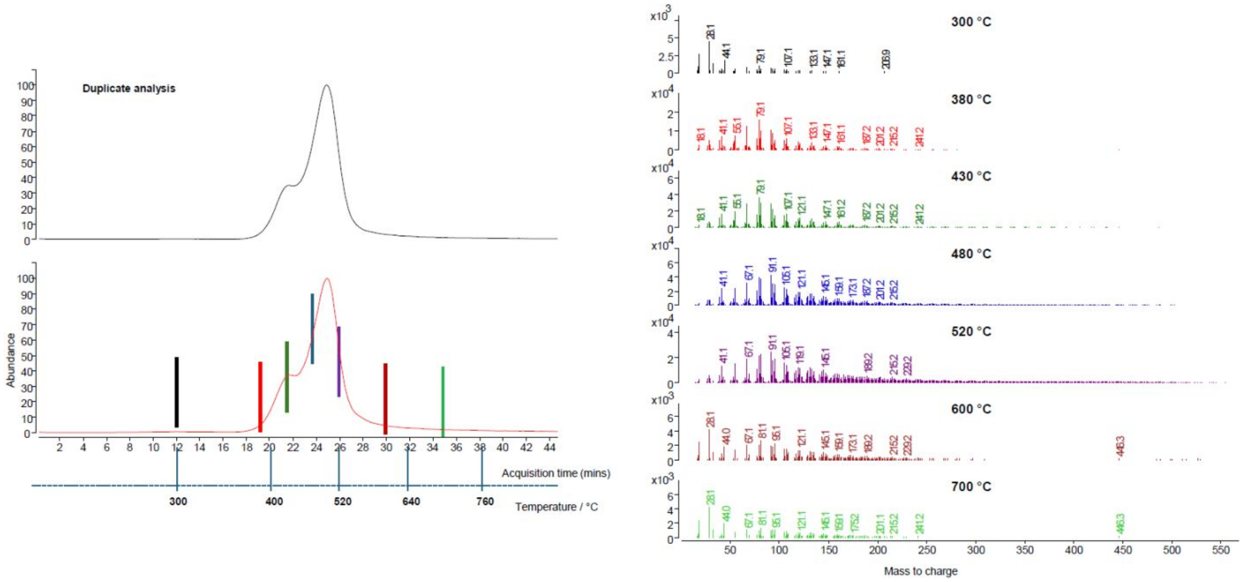

Figure S4a. EGA-MS thermograms and the corresponding MS scans obtained by HTPB heating from 100 to 900 °C with a gradient of 100 °C/min.

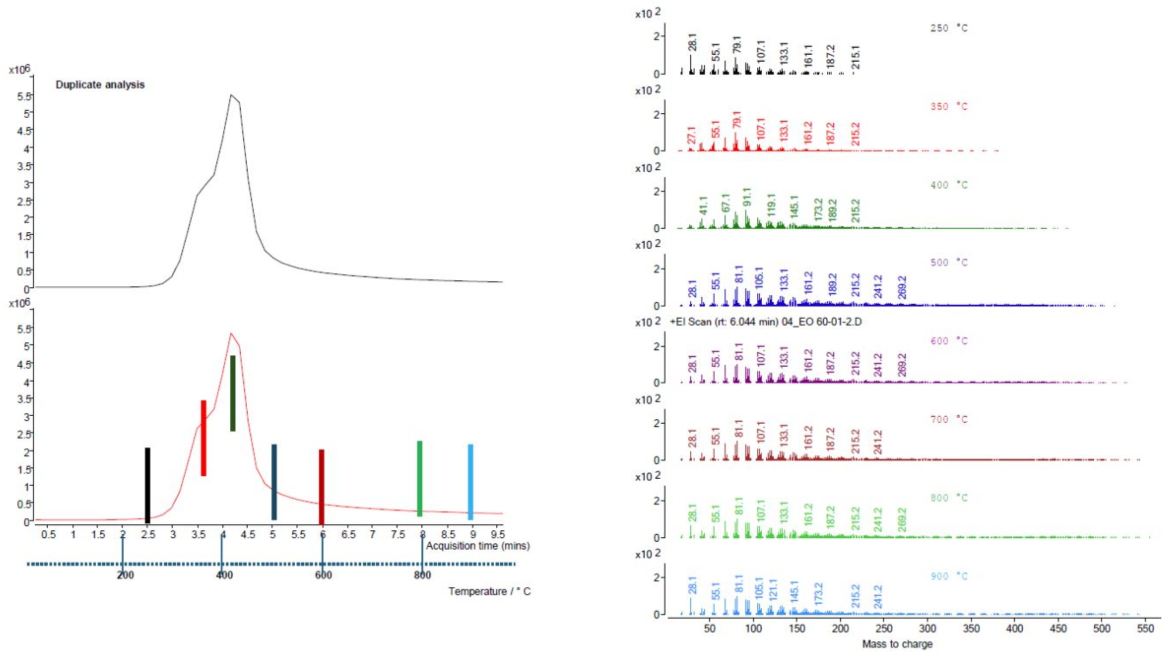

Figure S4b. EGA-MS thermograms and the corresponding MS scans obtained by HTPB heating from 100 to 900 °C with a gradient of 100 °C/min.

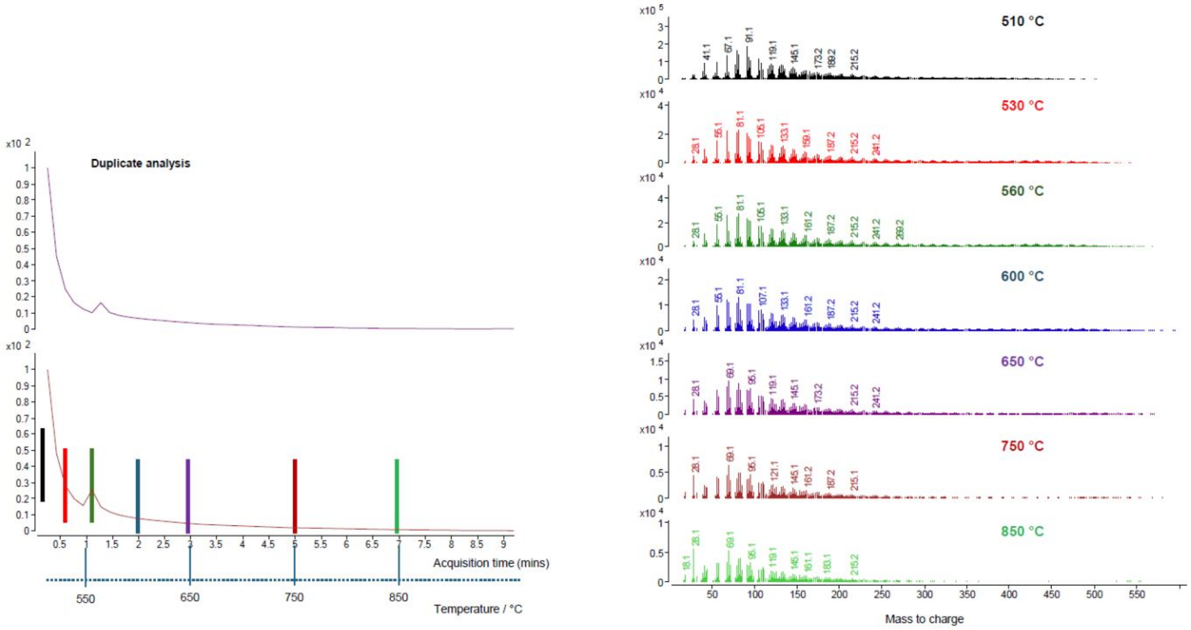

Figure S4c. EGA-MS thermograms and the corresponding MS scans obtained by HTPB heating from 500 to 1,000 °C with a gradient of 50 °C/min.

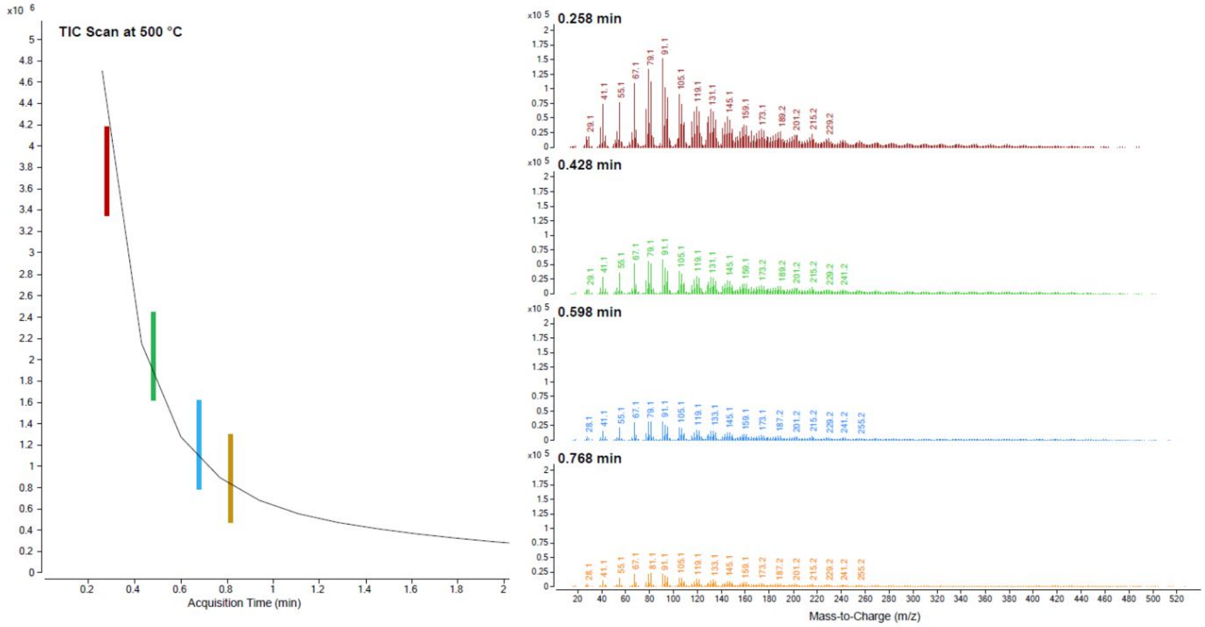

Figure S5a. EGA-MS thermograms and the corresponding MS scans using TIC (total ion current) showing no detectable presence of PAH ions at 500 °C.

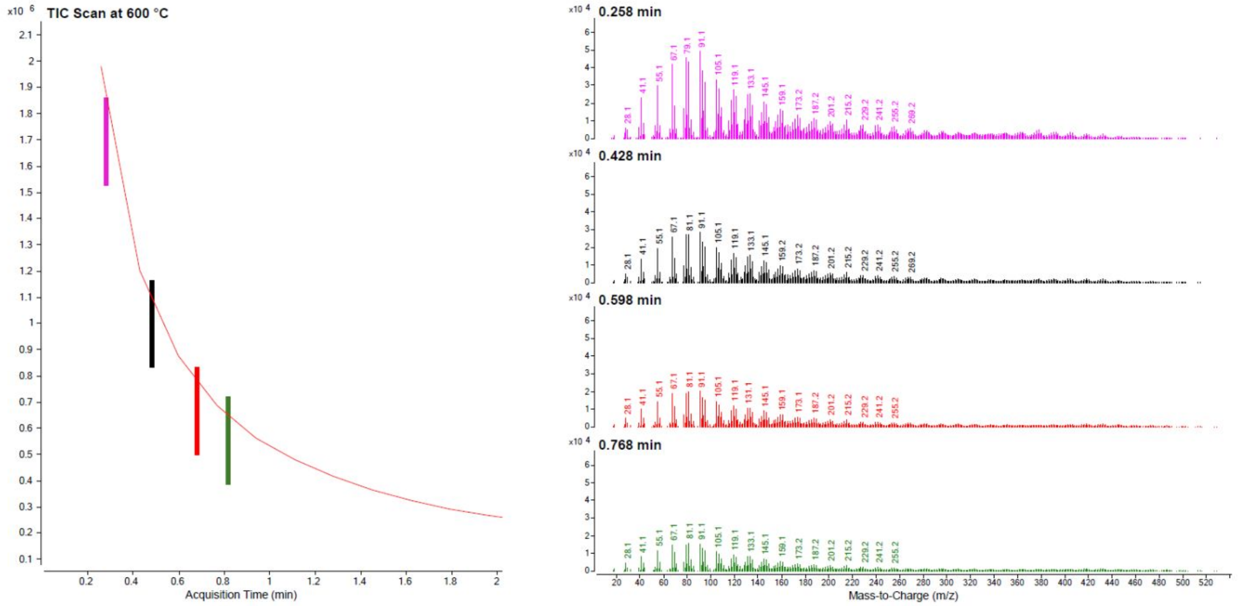

Figure S5b. EGA-MS thermograms and the corresponding MS scans usingTIC (total ion current) showing no detectable presence of PAH ions at 600 °C.

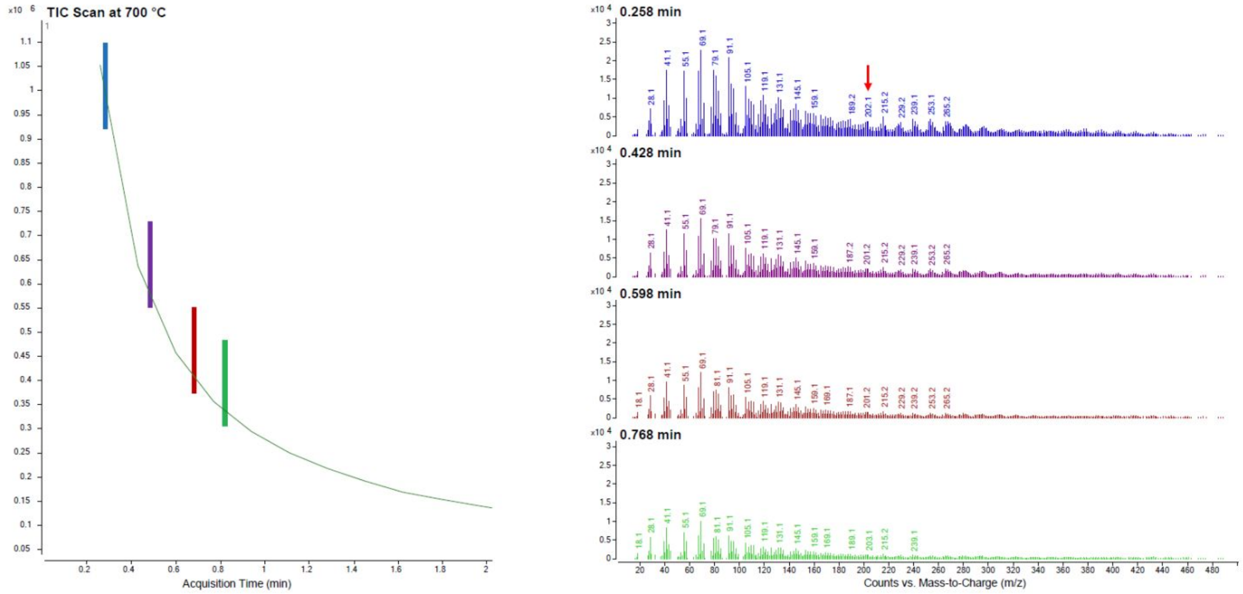

Figure S5c. EGA-MS thermograms and the corresponding MS scans using TIC (total ion current) showing trace presence of PAH ions at 700 °C.

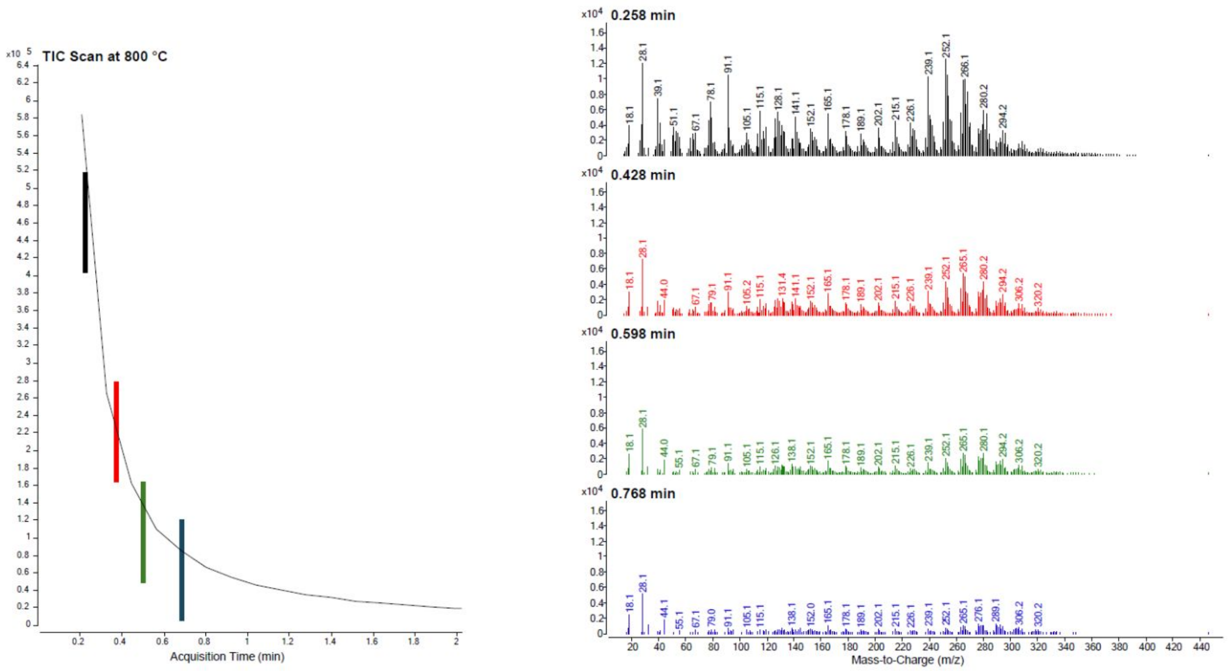

Figure S5d. EGA-MS thermograms and the corresponding MS scans using TIC (total ion current) showing the presence of PAH ions at 800 °C.

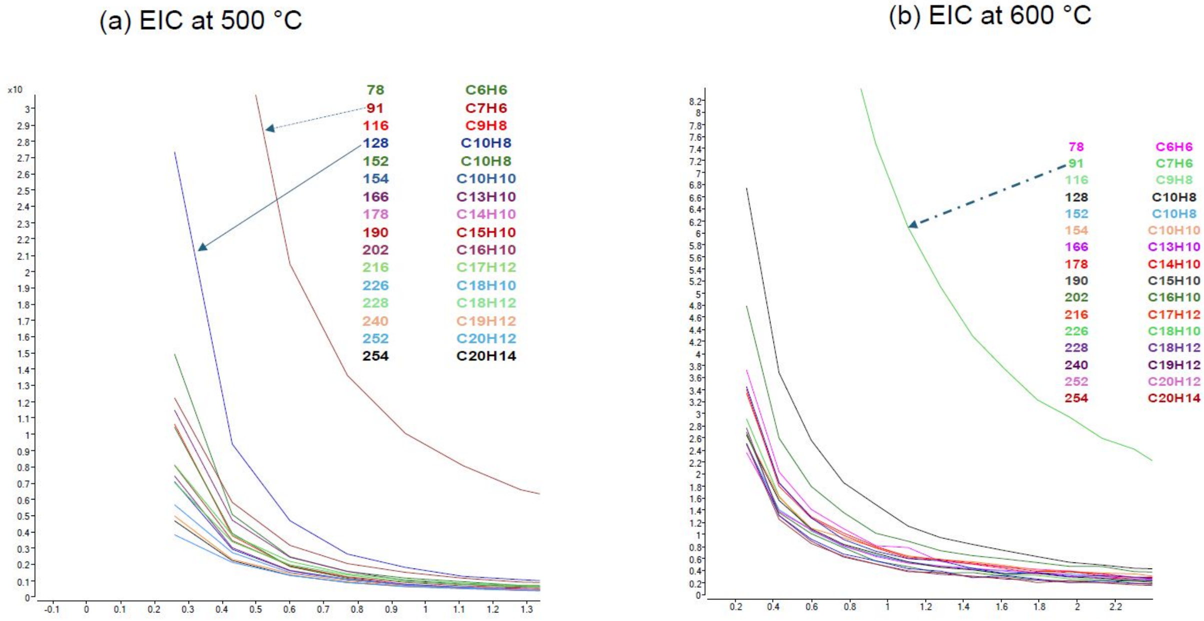

Figure S6a&b. EGA-MS thermograms and the corresponding MS scans using EIC (extracted ion current) showing a possible presence of PAH ions at 500 °C and 600 °C.

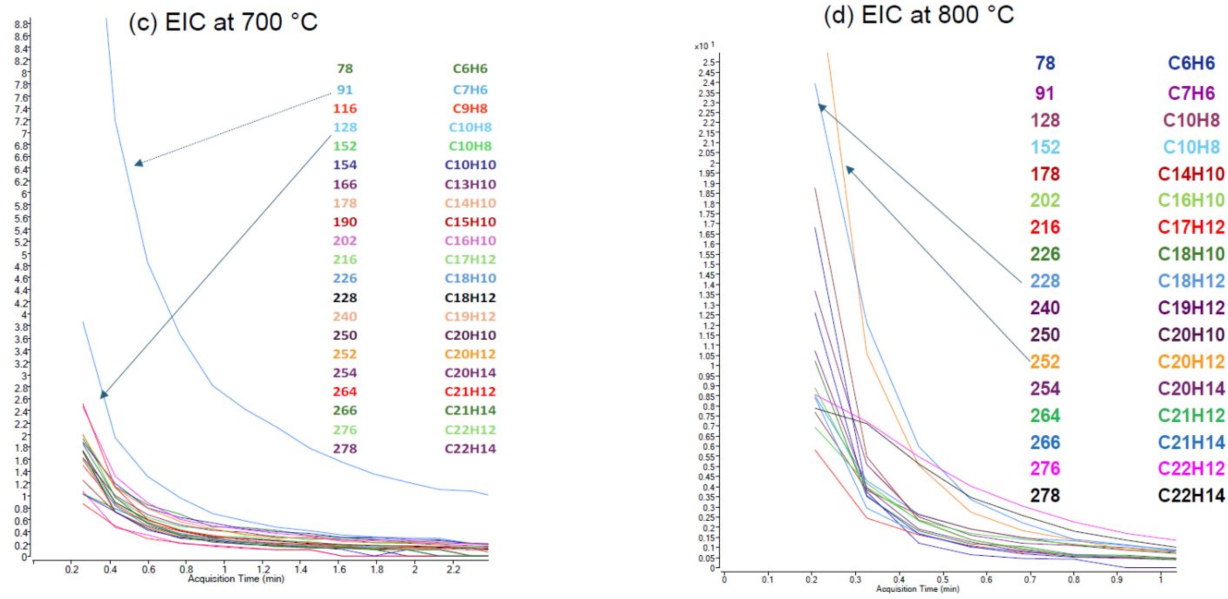

Figure S6c&d. EGA-MS thermograms and the corresponding MS scans EIC (extracted ion current) showing a possible presence of PAH ions at 700 °C and 800 °C.

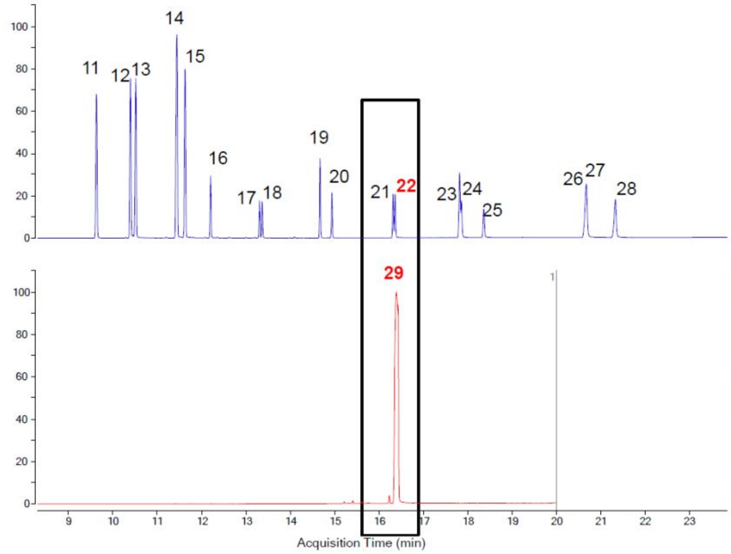

Peak	t_R	MW	Name of compound
11	9.62	128	Naphthalene
12	10.39	142	2-Methylnaphthalene
13	10.51	142	1-Methylnaphthalene
14	11.44	152	Acenaphthylene
15	11.62	154	Acenaphthene
16	12.20	166	Fluorene
17	13.31	178	Phenanthrene
18	13.36	178	Anthracene
19	14.67	202	Fluoranthene
20	14.94	202	Pyrene
21	16.31	228	Benz[*a*]anthracene
22	16.36	228	Chrysene
23	17.82	252	Benzo[*b*]fluoranthene
24	17.86	252	Benzo[*k*]fluoranthene
25	18.36	252	Benz[*a*]pyrene
26	20.64	278	Indeno[1,2,3-*cd*]pyrene
27	20.70	278	Dibenz[*a*,*h*]anthracene
28	21.32	276	Benzo[*g*,*h*,*i*]perylene
29	16.38	228	Triphenylene

Figure S7a. GC-MS chromatograms of PAH standards and the identification of peaks shown in the table.

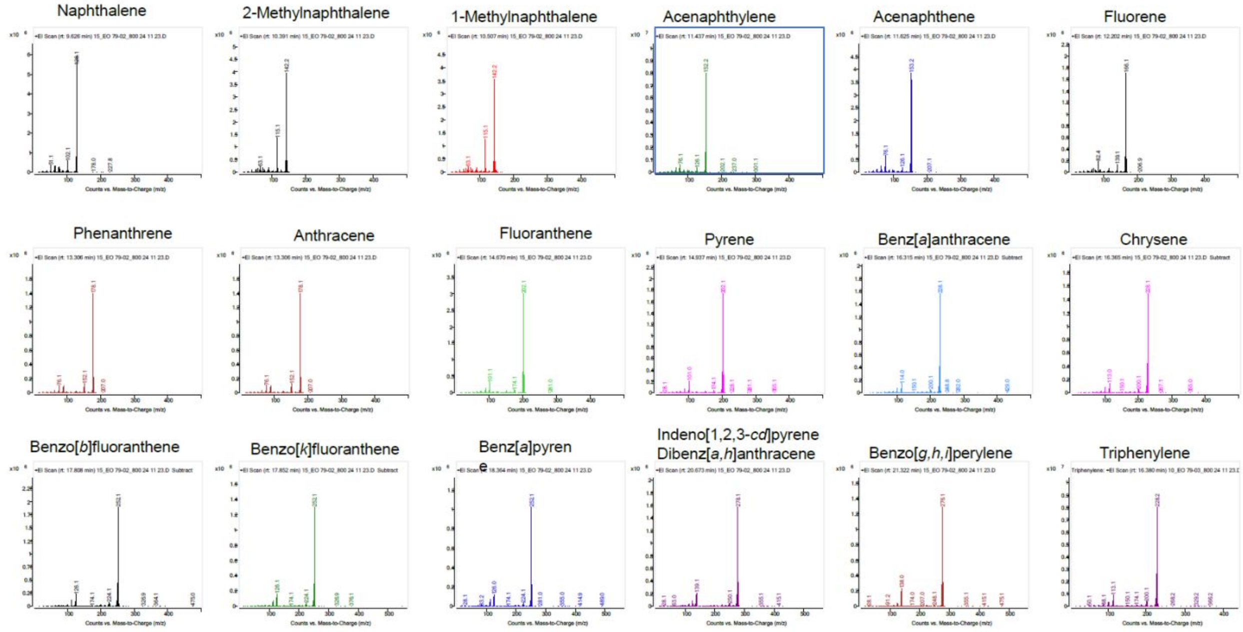

Figure S7b. MS spectra of PAH standards.

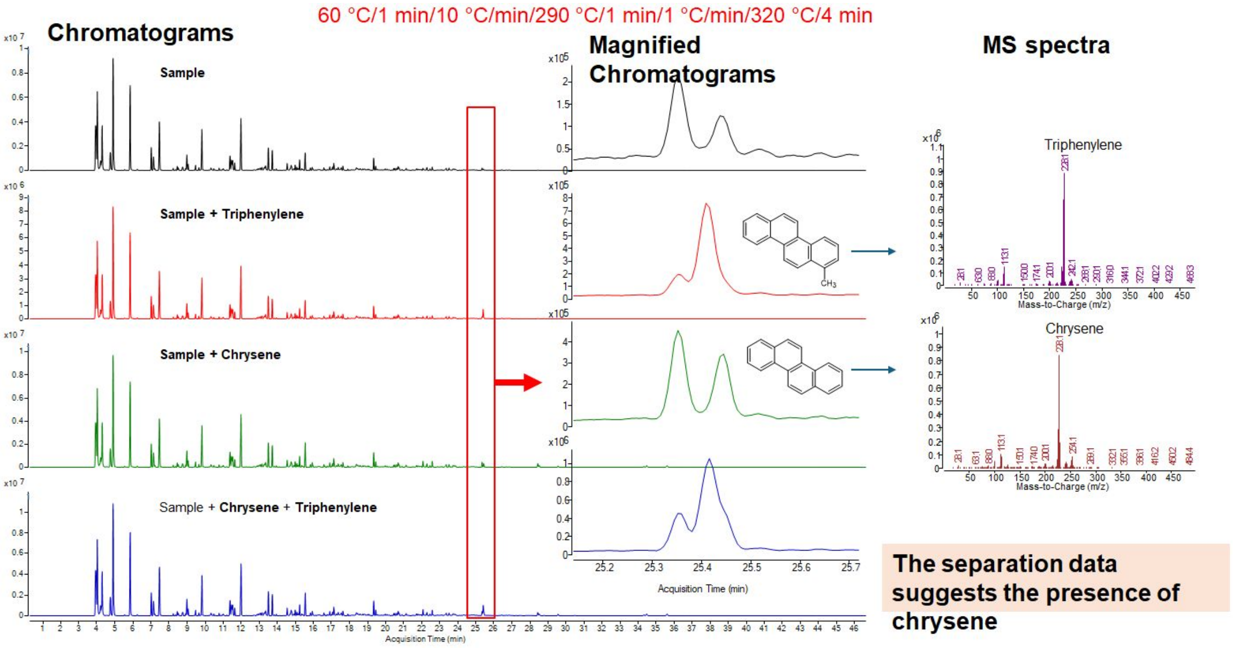

Figure S8. Application of standard addition method and optimized temperature ramp rate for chrysene identification.

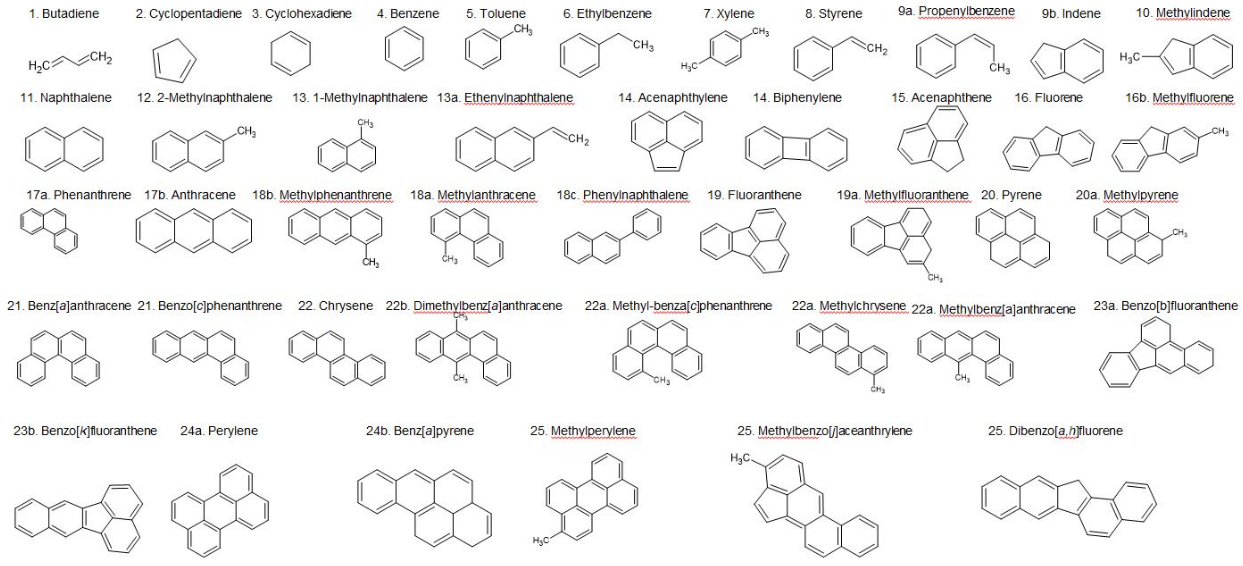

Figure S9. Chemical structures of compounds identified by Pyr-GC-MS.
